# Preferred orientations of garnet porphyroblasts reveal previously cryptic templating during nucleation

**DOI:** 10.1038/s41598-021-85525-7

**Published:** 2021-03-25

**Authors:** Alexandra B. Nagurney, Mark J. Caddick, David R. M. Pattison, F. Marc Michel

**Affiliations:** 1 Deparment of Geosciences, Virginia Tech, 926 West Campus Drive, Blacksburg, VA 24060 USA; 2 Department of Geoscience, University of Calgary, 2500 University Drive NW, Calgary, AB T2N 1N4 Canada

**Keywords:** Planetary science, Solid Earth sciences

## Abstract

Electron back scattered diffraction data of garnet crystals from the Nelson Aureole, British Columbia and the Mosher’s Island formation, Nova Scotia, reveals that 22 garnet crystals are all oriented with one of three crystal directions parallel to the trace of the foliation plane in thin section. Structural models suggest that these relationships are due to preferential garnet nucleation onto muscovite, with the alignment of repeating rows of Al octahedra and Si tetrahedra in each leading to inheritance of garnet orientation from the muscovite. These results highlight that epitaxial nucleation may be a prevalent process by which porphyroblast minerals nucleate during metamorphism and carry implications for the role that non-classic nucleation pathways play in the crystallization of metamorphic minerals, the distribution of porphyroblasts in metamorphic rocks, and, in cases in which nucleation is the rate limiting step for crystallization, the energetics of metamorphic reactions.

## Introduction

Deciphering the mechanisms of mineral crystallization has important implications for understanding many geologic processes. Classical theory states that nucleation and growth are each a single step process based on monomer-by-monomer addition of simple chemical species^1^. However, this model may not be appropriate for many geologic systems, and minerals may crystallize via multi-step non-classical pathways, such as the formation of intermediate nanocrystals, amorphous nanoparticles, or via nucleation on a substrate^2^. Two common examples of non-classical nucleation pathways are epitaxy, which is when a phase overgrows a substrate, and topotaxy, in which a reactant phase is converted into a new product phase that utilizes part of the precursor structure^3^.

Epitaxy and topotaxy have been previously identified in a range of geologic settings. In igneous systems, epitaxial nucleation may influence alignment and fabrics of ferromagnetic minerals and influence magma composition evolution^4^. In both calcareous deep sea sediments and high pressure eclogite facies rocks, seismic anisotropy may be enhanced by the oriented crystallization of minerals^5, 6^. During continental collision, the products of dehydration melting reactions may preferentially crystallize on certain phases^7^. Some readily identified forms of epitaxy during prograde metamorphism include sillimanite nucleation on biotite, staurolite on kyanite, sillimanite on andalusite, and K-feldspar on albite^8–11^. It is currently unclear whether epitaxial nucleation is a more widespread process that also controls the development of rocks without clearly preserved evidence of such templating.

Garnet is an important and widespread metamorphic mineral, with a composition that is sensitive to changing pressure–temperature (*P–T*) conditions, hence its common use in quantitative thermobarometry^12^. It can be dated using multiple isotopic systems, which can be coupled with thermodynamic models to understand the rates of tectonic processes^13^ and with stable isotope studies to reveal records of fluid-rock interaction in the crust^14^. Despite its clear petrologic utility, details of the atomic-scale processes by which garnet crystallizes (nucleates and grows), and their controls on suitable nucleation sites, growth rates, and the apparent overstepping of reactions in which garnet is a product phase, are poorly constrained. Here, we utilize garnet crystal orientation data and models for the atomic structure of garnet, chlorite, and muscovite to explore whether initial garnet crystallization inherits aspects of pre-existing mineral phases, thus biasing crystal growth to specific textural locations within metamorphic rocks.

Despite its cubic crystal structure and common form as a porphyroblast, garnet has been previously identified to crystallize via epitaxial and topotaxial relationships with muscovite, biotite, and pyroxene. This manifests as the parallelism of high symmetry crystal planes in one phase to those in another phase^15, 16^ with the most commonly reported relationship being {1 1 0}_garnet_ // (0 0 1)_mica_ (where ‘//’ = ‘parallel’ and (hkl) are crystal planes)^16–19^, though others have also been identified^20–22^. However, these examples all either represent unusual microstructures, such as atoll or snowball garnets^21, 22^, or formed at extreme environments such as ultra-high pressure metamorphism^17^. There is currently little understanding of whether epitaxial and/or topotaxial nucleation processes occur more broadly during regional and contact metamorphism of less exotic, foliated, pelitic rocks. More generally, it is unclear whether these processes are important for, or potentially control, porphyroblast crystallization. Deciphering whether epitaxial and/or topotaxial nucleation may play a role in more typical metamorphic rocks, where ‘unusual’ growth habits are absent is important to elucidate which kinetic factors may be the rate-limiting step for mineral crystallization.

## Results

### Sample description

We investigated whether epitaxial and/or topotaxial nucleation played a role in garnet crystallization in three samples from two localities: the garnet zone of the Nelson contact aureole, British Columbia (samples 08-CW-7.5 and 08-CW-7A)^23, 24^, and the staurolite grade Mosher’s Island formation, Nova Scotia (sample 2018PPGrt_01)^25, 26^ (Supplemental Figs. S1–3). All three samples contain biotite, chlorite, garnet, muscovite, plagioclase, and quartz. In each case, garnet overgrows a foliation which is defined by the shape preferred orientation of prograde chlorite and muscovite. This persistence of primary chlorite and muscovite is consistent with calculated phase assemblages for the Nelson aureole samples at apparent peak temperatures of ~ 530 °C and 3.5 kbar^24^ and for the Mosher’s Island formation sample at 550 ºC and 4.1 kbar^27^. Sample 2018PPGrt_01 also contains staurolite and late-stage chlorite overgrowths that are texturally distinct from the primary chlorite (Supplemental Fig. S3). Thin sections of the samples were cut perpendicular to the rock foliation and lineation.

### Electron back scattered diffraction analysis

**Figure 1:**
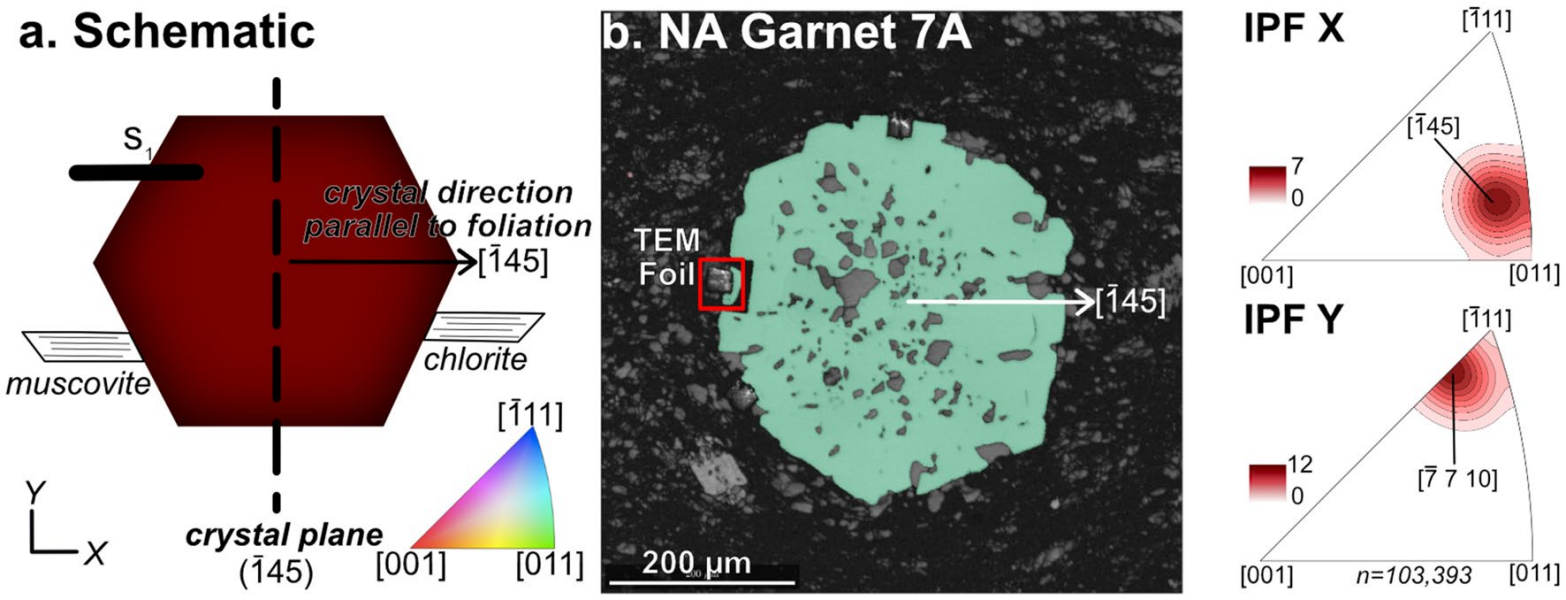
EBSD results. (**a**) Plotting schematic of garnet in B, showing garnet crystal direction and corresponding crystal plane. The trace of the S_1_ foliation in the thin section is parallel to the horizontal (X) direction. XY coordinates are defined here as X: parallel to the horizontal direction of the thin section plane (parallel to foliation), Y: parallel to the vertical direction of the thin section plane (perpendicular to foliation). (**b**) Electron back scattered diffraction (EBSD) analyzed garnet crystal, color-coded for crystal direction parallel to the X direction and the S_1_ foliation (color coding following inset in (**a**)). Location of TEM foil (Fig. 2) is shown. TEM foil was cut perpendicular to (into) the thin section plane. Orientation data are plotted on the EBSD band contrast image. Inverse pole figures (IPF) X and Y show the crystal directions of garnet that are parallel (X) and perpendicular (Y) to the foliation. IPFs are contoured for multiples of uniform distribution.

We analyzed the crystallographic orientation of seven garnet crystals from the Nelson Aureole (NA) and fifteen crystals from the Mosher’s Island Formation (MI) with Electron Back Scattered Diffraction (EBSD). The plotting schematic for all EBSD images is shown in Fig. 1a, with EBSD data for a representative garnet crystal shown in Fig. 1b. Data for the remaining crystals are shown in the Supplemental Figs. S4–5, and Supplemental Table 1, with the trace of the primary foliation (which is defined by the shape preferred orientation of muscovite and chlorite in all samples) parallel to the horizontal direction of the thin section plane in each case. Garnet is color coded for the crystal direction that is parallel to the trace of the S_1_ foliation of the rock in the thin section plane shown in the IPF color scheme in the inset. For the representative sample shown in Fig. 1b, (NA Garnet 7A), [$\stackrel{-}{1 }4 \,5$]_gt_ and [$\stackrel{-}{7} 7\, 10$]_gt_ are parallel and perpendicular to the foliation, respectively.

### Transmission electron microscopy

**Figure 2:**
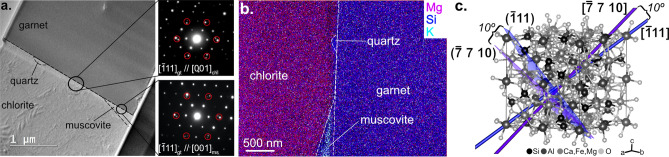
TEM results. (**a**) Transmission Electron Microscopy (TEM) image of mineral interface. Diffraction patterns of the garnet-chlorite interface (top) and garnet-muscovite interface (bottom) oriented such that [$\stackrel{-}{1} 1\, 1$]_gt_ is parallel to [0 0 1]_chl,ms_. The doubling of planes (highlighted by red circles) suggests an epitaxial relationship between garnet and chlorite and between garnet and muscovite. (**b**) Stacked electron dispersive spectrometry maps of Mg (magenta), Si (blue), and K (light blue) abundances showing the 4 phases in the TEM foil. Sample is rotated relative to (**a**). (**c**) Garnet crystal structure (from Novak and Gibbs^28^) highlighting the [$\stackrel{-}{1} 1 \,1$]_gt_ and [$\stackrel{-}{7} 7 \,10$]_gt_ crystal directions (blue and purple lines, respectively) and ($\stackrel{-}{1} 1 \,1$)_gt_ and ($\stackrel{-}{7} 7 \,10$)_gt_ planes (blue purple sheets).

A garnet-chlorite and garnet-muscovite interface from NA Garnet 7A was investigated at higher resolution by Transmission Electron Microscopy (TEM) so that the nanoscale structure of the grain boundary could be compared to the larger area EBSD orientation results. The location of the TEM foil is shown in Fig. 1b and Supplemental Fig. S6. In Fig. 2a, the smaller ‘wedge’ shaped muscovite crystal (compared to the larger chlorite) is a function of the TEM foil preparation. The muscovite grain extends beyond the prepared foil, but was truncated due to the small (10 µm × 10 µm) foil size. In this orientation, (0 0 1)_chl_ // (0 0 1)_ms_ (Supplemental Fig. S7). Figure 2a shows that the interface is a planar surface without void spaces. The sharpness of the diffraction patterns indicates that both phases are crystalline, with diffraction patterns of the garnet-chlorite and garnet-muscovite interfaces (located with black circles in Fig. 2a) revealing a doubling of the (0 0 1)_chl,ms_ and ($\stackrel{-}{1} 1 \, 1$)_gt_ planes (red circles). This indicates that the (0 0 1)_chl,ms_ and ($\stackrel{-}{1} 1 \,1$)_gt_ planes are parallel to each other, which can be interpreted as an epitaxial/topotaxial relationship in which [0 0 1]_chl,ms_ is parallel to [$\stackrel{-}{1 }1 \,1$]_gt_. Compositional analyses reveal that, in addition to muscovite at the grain boundary, there is also a nano-sized quartz grain at this interface (Fig. 2b).

Comparison of this relationship apparent at the nanoscale (Fig. 2) with EBSD results for the same garnet crystal (Fig. 1) reveals consistency between the interpretation of the two datasets. The EBSD and TEM results show that [$\stackrel{-}{7} 7 \,10$]_gt_ and [$\stackrel{-}{1} 1 \,1$]_gt_ are perpendicular to the foliation, respectively. Plotting these directions and their corresponding planes on the crystal structure of garnet^28^ (lines and rectangular planes, respectively, in Fig. 2c), reveals that these orientations are within 10º of each other. Therefore, the macroscale orientations observed via EBSD for a relatively large population of garnet crystals are consistent with nanoscale TEM observations of the mineral interface.

### Synthesis of results for 22 garnet crystals

**Figure 3:**
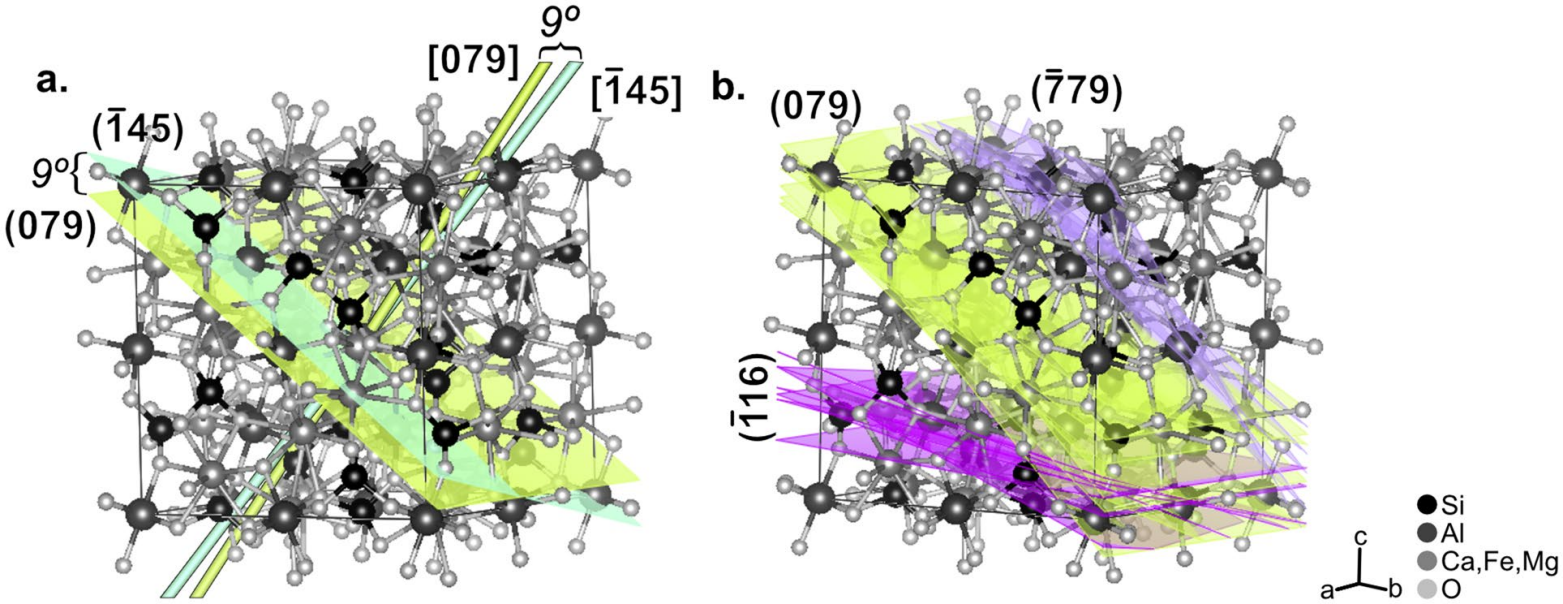
Summary of EBSD results. (**a**) Garnet crystal structure showing the [$\stackrel{-}{1} 4 \, 5$]_gt_ crystal direction (teal line) and ($\stackrel{-}{1} 4 \,5$)_gt_ crystal plane (teal line and sheet), and the [0 7 9] direction and plane (lime green line and sheet). ($\stackrel{-}{1} 4 \,5$)_gt_ and (0 7 9)_gt_ have 9º angular misorientation. See text for explanation. (**b**) Garnet crystal structure showing the planes corresponding to the 22 crystal directions of garnet that were found to be parallel to the trace of the foliation in thin section. Planes cluster into three groups centered around ($\stackrel{-}{1} 1 \,6$) (pink), (0 7 9) (green), and ($\stackrel{-}{7} 7 \,9$) (purple).

The orientations of all 22 analyzed garnet crystals were plotted on crystal structure models of garnet^28^. Figure 3a shows the [$\stackrel{-}{1}4 \, 5$]_gt_ crystal direction (teal arrow), which is oriented parallel to the trace of the foliation in thin section in Fig. 1, and the corresponding ($\stackrel{-}{1}4 \, 5$)_gt_ crystal plane (teal plane) plotted on the crystal structure model. This demonstrates that it is more illustrative to utilize crystal planes (instead of vector directions) to study relationships within the atomic structure of garnet. Accordingly, although our EBSD results are initially recorded as crystal directions parallel to the trace of the foliation, we show the corresponding planes (the poles to these directions) for all 22 garnet crystals on the garnet structure model in Fig. 3b.

The orientations of all 22 analyzed garnets fall into just three clusters: nine are oriented within 13º of the ($\stackrel{-}{1} 1 \,6$) plane (pink in Fig. 3b), eight are within 14º of the (0 7 9) plane (green), and five are within 12º of the ($\stackrel{-}{7} 7 \,9$) (purple) plane (Fig. 3b). As an example, Fig. 3a shows both the [$\stackrel{-}{1} 4 \, 5$]_gt_ and [0 7 9]_gt_ crystal directions (teal and lime green arrows), and the corresponding ($\stackrel{-}{1} 4 \, 5$)_gt_ and (0 7 9)_gt_ crystal planes (teal and lime green surfaces), highlighting that ($\stackrel{-}{1} 4 \, 5$)_gt_ is only 9º from the (0 7 9)_gt_, which is the average crystal orientation for that cluster of planes.

The results are summarized as nine garnet crystals oriented with [$\stackrel{-}{1} 1 \,6$]_gt_ approximately parallel to the trace of the foliation in thin section, eight in which [0 7 9]_gt_ is parallel to the foliation, and five in which [$\stackrel{-}{7} 7 \,9$]_gt_ is parallel to the foliation. Both (0 7 9)_gt_ and ($\stackrel{-}{7} 7 \,9$)_gt_ are close to low index planes of garnet, with (0 7 9)_gt_ having a 7º angular misorientation from (0 1 1)_gt_ and ($\stackrel{-}{7} 7 \,9$)_gt_ having a 7º angular misorientation from ($\stackrel{-}{1} 1 \,1$)_gt_. ($\stackrel{-}{1} 1 \,6$)_gt_ is not close to low index planes of garnet. Further, there are only four crystal directions: [$\stackrel{-}{3} 4 \,6$]_gt_, [$\stackrel{-}{2} 11 \,11$]_gt_, [$0 1 2$]_gt_, and [$\stackrel{-}{7} 7 \,10$]_gt_, that are perpendicular to the foliation of the samples (Supplemental Table S1). As such, there is also a clustering in which {$\stackrel{-}{3} 4 \,6$}_gt,_ {$\stackrel{-}{2} 11 \,11$}_gt,_ {0 1 2}_gt_, or {$\stackrel{-}{7} 7 \,10$}_gt_ are preferentially parallel to (0 0 1)_ms,chl_.

## Discussion

The relationship between garnet crystal orientation and rock foliation shown in Fig. 3b requires an assessment of its potential crystallographic controls. Muscovite and chlorite define the rock foliation so it is possible that garnet will template on the crystal structure of one or both of these minerals. Al octahedra and Si tetrahedra are the building blocks of the garnet structure, so templating on these structures seems most likely. Here, we investigate the crystal structures of both chlorite and muscovite to determine which mineral’s crystal structure contains elements that may be advantageous for garnet to template on.

**Figure 4:**
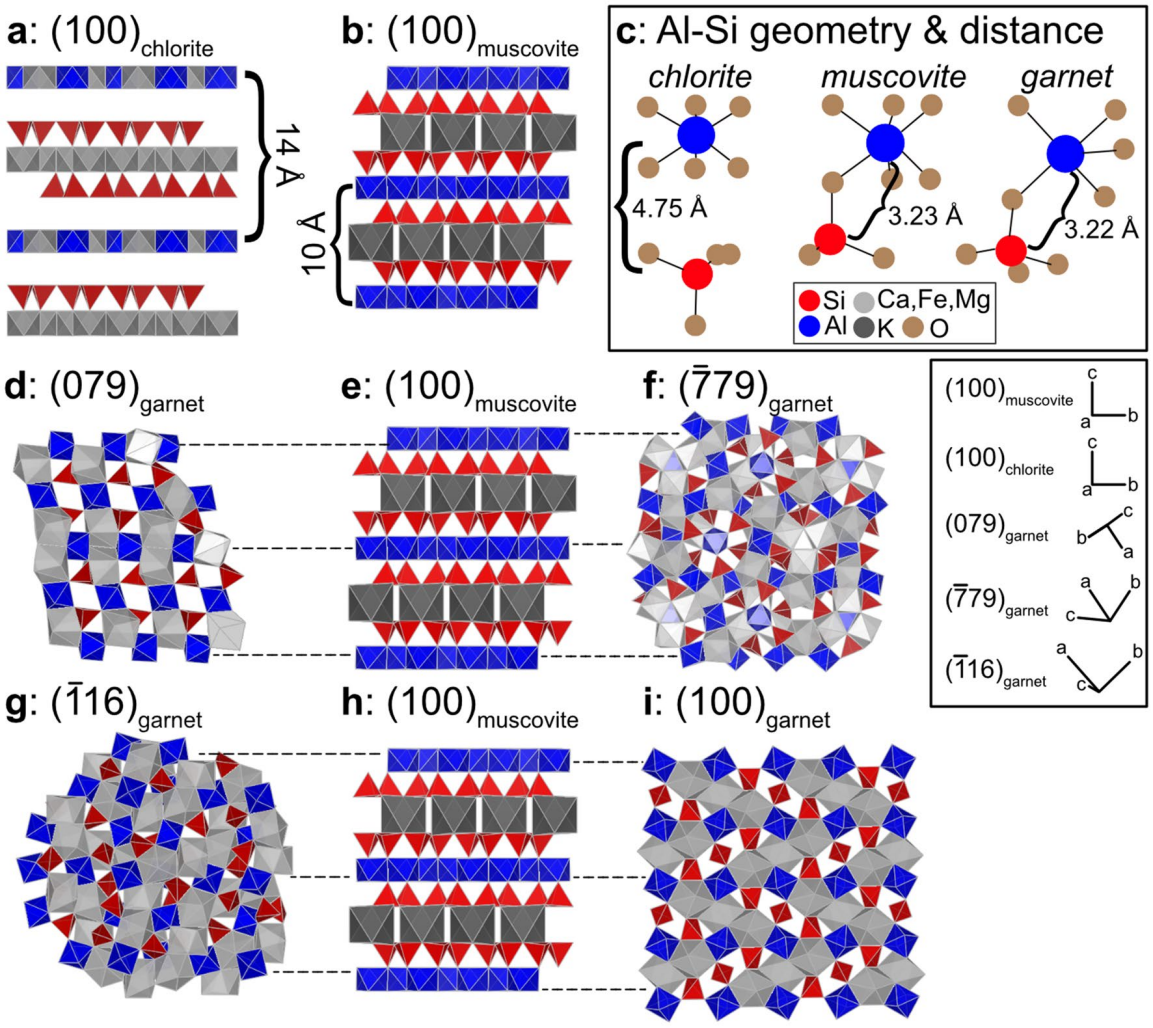
Crystal structure models of (**a**) chlorite^48^ and (**b**) muscovite^30^. (**c**) Al octahedra–Si tetrahedra geometries and distances in chlorite, muscovite, and garnet. (**d**–**i**) Crystal structure models of (0 7 9)_gt,_ (100)_ms_, ($\stackrel{-}{7} 7 \,9$)_gt,_ ($\stackrel{-}{1 }1 \, 6$)_gt_, (1 0 0)_ms_, and (1 0 0)_gt_, with dashed lines highlighting relationships between Al in each. The inset shows the 3D orientation of all of the crystal planes.

Despite both being sheet silicate minerals, the types of bonds, geometries, and interatomic distances between Al and Si are different in chlorite and muscovite. In chlorite, the sheet layers are bonded together via hydrogen bonds and van der Waals forces, while in muscovite the sheets are connected via bridging oxygens (Fig. 4a,b)^29, 30^. In chlorite, Al occurs either in the interlayer octahedral sheet or substitutes for Si in the tetrahedral layer (Fig. 4a)^29, 31^. In muscovite, Al is in the octahedral layer and connected to the Si tetrahedral layer via bridging oxygens (Fig. 4b)^30^. Further, the geometric arrangements of Al and Si are different in chlorite and muscovite (Fig. 4c). Finally, the distance between Al and Si in chlorite is 4.75 Å, while in muscovite the distance between the cations is 3.23 Å (Fig. 4c)^29, 30^.

**Table 1:** Distances between and along Al and Si rows in chlorite, garnet, and muscovite.

Distance between Al ‘rows'		Distance between Si ‘rows'		Al-Al distances along the row		Si–Si distances along the row			Si–O–Al distance	
(1 0 0)_chl_	14.38 Å	(1 0 0)_chl_	8.97 Å	(1 0 0)_chl_	9.23 Å	(1 0 0)_chl_	3.07 Å	6.16 Å	(1 0 0)_chl_	4.75 Å
(1 0 0)_ms_	10.12 Å	(1 0 0)_ms_	5.64 Å	(1 0 0)_ms_	6.03 Å	(1 0 0)_ms_	2.97 Å	6.06 Å	(1 0 0)_ms_	3.23 Å
(0 7 9)_gt_	9.56 Å	(0 7 9)_gt_	5.39 Å	(0 7 9)_gt_	4.99 Å	(0 7 9)_gt_		5.39 Å	(0 7 9)_gt_	3.22 Å
($\stackrel{-}{7} 7\, 9$)_gt_	9.99 Å	($\stackrel{-}{7} 7 \,9$)_gt_	5.39 Å	($\stackrel{-}{7} 7 \,9$)_gt_	4.99 Å	($\stackrel{-}{7} 7 \,9$)_gt_	3.53 Å		($\stackrel{-}{7} 7 \,9$)_gt_	3.22 Å
($\stackrel{-}{1 }1 \,6$)_gt_	8.15 Å	($\stackrel{-}{1 }1 \,6$)_gt_	5.39 Å	($\stackrel{-}{1 }1 \,6$)_gt_	4.99 Å	($\stackrel{-}{1 }1 \,6$)_gt_		5.39 Å	($\stackrel{-}{1 }1 \,6$)_gt_	3.22 Å
(1 0 0)_gt_	11.53 Å	(1 0 0)_gt_	X	(1 0 0)_gt_	5.77 Å	(1 0 0)_gt_	X	X	(1 0 0)_gt_	3.22 Å

All measurements were made using VESTA^46^.

The geometry and distance between Al and Si in muscovite are similar to that in garnet, with 3.23 Å between Al and Si atoms in muscovite and 3.22 Å in garnet (Fig. 4c)^28, 30^. Any section of muscovite oriented as shown in Fig. 4b will expose Al-Si frameworks that could theoretically be adopted by the garnet crystal structure, providing potentially preferable nucleation sites. As such, due to the similarities between: (i) interatomic distances and (ii) Al-Si geometries in muscovite and garnet (that are lacking between garnet and chlorite), we interpret that it is more likely for garnet to template on the crystal structure of muscovite than chlorite. As such, we focus here on how elements of the garnet structure in the (0 7 9)_gt_ and ($\stackrel{-}{7} 7 \,9$)_gt_, and ($\stackrel{-}{1} 1 \,6$)_gt_ planes may align with that of muscovite.

Figure 4d–i shows how the three garnet orientations found to be parallel to the trace of the foliation in our dataset may template on to the muscovite crystal structure, with Supplemental Videos S1–3 showing these relationships in the third dimension. In these three orientations, multiple horizontal ‘rows’ of Al atoms in garnet are separated by distances that are very similar to the stacking distances of corresponding Al sheet-like layers in muscovite (these rows are annotated by dashed lines between Fig. 4d–f and g–i). Furthermore, the distances between Si sheet-like layers in muscovite corresponds well with equivalent ‘rows’ of Si tetrahedra in garnet in those orientations (Table 1), as does the distance between Al and Si atoms within each row. These relationships are clearer for (0 7 9)_gt_ and ($\stackrel{-}{7} 7 \,9$)_gt_, than ($\stackrel{-}{1} 1 \,6$)_gt_ (Fig. 4).

For comparison, (1 0 0)_gt_ is shown in Fig. 4i. Rows of Al octahedra in muscovite and garnet can be matched. However, there are no corresponding ‘rows’ of Si tetrahedra in garnet with similar distances to those in muscovite. Since (1 0 0)_gt_ lacks this similarity with (1 0 0)_ms_, we infer that garnet is less likely to template onto muscovite in this orientation. This highlights that certain crystal planes of garnet share more similarities with aspects of (1 0 0)_ms_, implying a control for the relationships determined by EBSD. The clear similarities in bond geometry and interatomic spacings between muscovite and the observed garnet orientations suggest that it is likely that muscovite provides an ideal structure for garnet to template onto. This nucleation likely occurs across the terminations of the muscovite crystals with garnet oriented in one of the three orientations discussed above. Further, the prevalence of muscovite in these rocks suggests that muscovite provides plentiful nucleation sites on which garnet can nucleate during prograde metamorphism. As such, the crystallographic relationship between garnet and chlorite shown in Fig. 2a may be coincidental rather than genetic: muscovite and chlorite are generally sub-parallel in these samples (Supplemental Figs. S3–7), so inheritance of garnet orientation from muscovite resulted in parallelism with chlorite.

**Figure 5:**
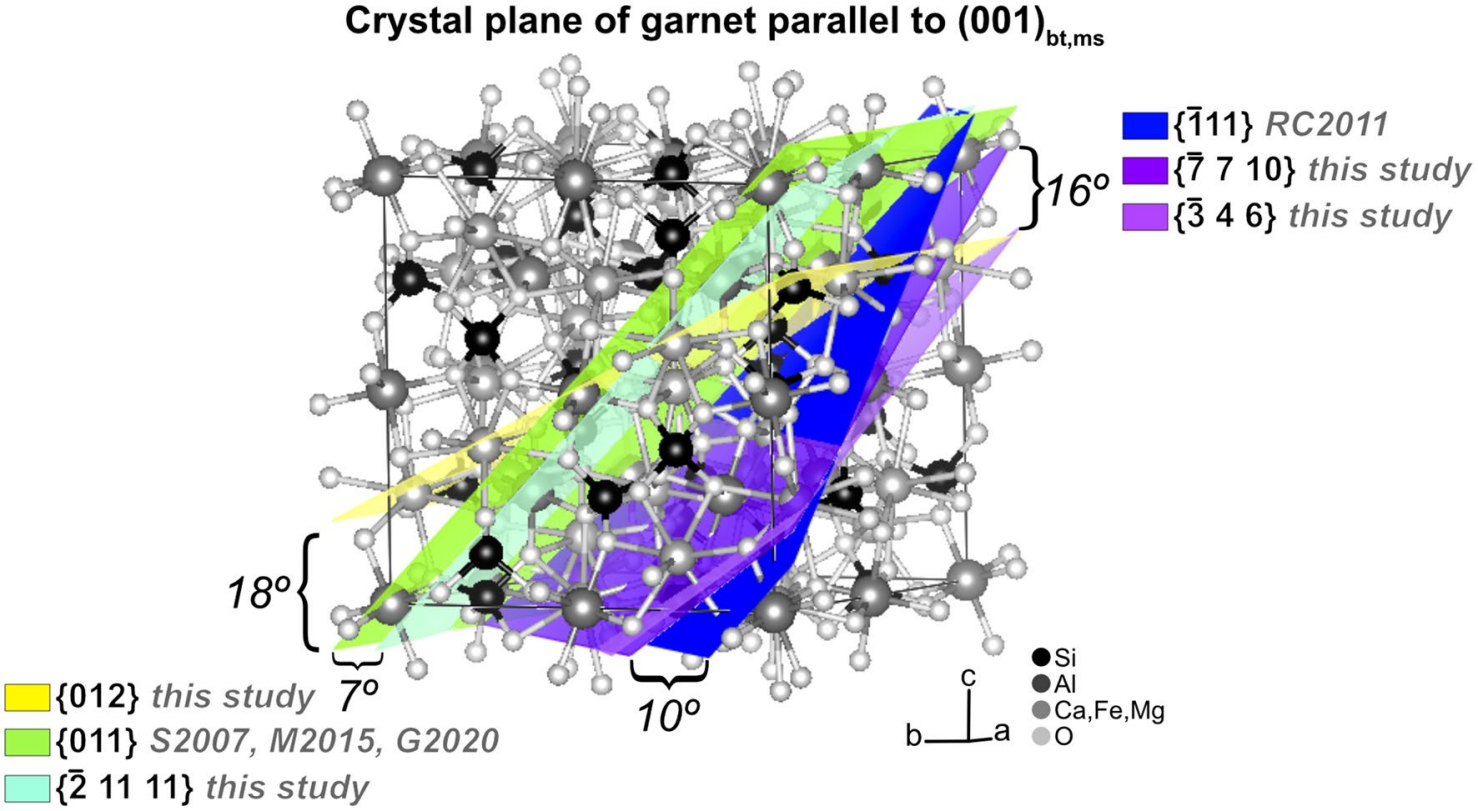
Comparison of crystal planes discussed in this study with others previously described as being preferentially parallel to (0 0 1)_bt,ms_ [RC2011 = Ruiz Cruz, (2011), S2007 = Spiess et al., (2007), M2015 = Moore et al., (2015), and G2020 = George and Gaidies, (2020)].

These interpretations agree well with previous studies that interpret that garnet can nucleate on the crystal structure of muscovite and/or biotite^16–18, 21^, with these previous studies showing (1 1 0)_gt_ or ($\stackrel{-}{1} 1 \,1$)_gt_ being within 20° of parallel to (0 0 1)_ms,bt_. Our results show a clustering in which one of ($\stackrel{-}{3} 4 \,6$)_gt,_ ($\stackrel{-}{2} 11 \, 11$)_gt,_ (0 1 2)_gt_, or ($\stackrel{-}{7} 7 \, 10$)_gt_ are preferentially parallel to (0 0 1)_ms_. These crystal planes are plotted on the crystal structure of garnet in Fig. 5, illustrating that ($\stackrel{-}{3} 4 \,6$)_gt_ and ($\stackrel{-}{7} 7 \,10$)_gt_ are within 16° of ($\stackrel{-}{1} 1\, 1$)_gt_ and that ($\stackrel{-}{2} 11 \,11$)_gt_ and (0 1 2)_gt_ are 7° and 18° from (0 1 1)_gt_, respectively. This generally agrees with previous results^16–18, 21^, suggesting that garnet nucleates epitaxially on the crystal structure of muscovite and/or biotite in the more exotic microstructures presented in those studies. Our results and interpretations further show that epitaxial nucleation is not restricted to unusual microstructures, but instead may be important during crystallization of many or most regional and contact metamorphic rocks.

An alternative atomic scale model for epitaxial nucleation of garnet on biotite involves garnet nucleation on distorted pseudo-hexagonal oxygen ring structures on (0 0 1)_bt_ surfaces^17^. This requires the addition of Al and extra O atoms to the biotite ring structure, driving distortion of the pseudo-hexagonal rings, and providing the surface for which garnet can template onto. In comparison, our mechanism of garnet templating does not require any addition or movement of atoms to the muscovite crystal lattice, potentially implying a more favorable mechanism for garnet to nucleate, though both mechanisms of templating are likely possible. More work modelling the atomic scale energetic interactions between these minerals is necessary to resolve these different interpretations.

From a different perspective, energetically favorable nucleation sites for garnet in a natural rock include crystal dislocations^32^ and locations of elevated HREE + Y concentrations^33^. Even in these situations, the garnet that grows may template onto nearby muscovite grains. Our study also supports the model in which garnet nucleation is controlled by a preexisting fabric and garnet first nucleates at grain boundaries in mica-rich layers^34, 35^. Garnet thus likely preferentially nucleates at the edge of muscovite crystals, adopting a specific crystal orientation, with grain boundaries providing efficient transport of nutrient elements to the growing nucleus.

The interpretations of epitaxial nucleation presented here and in previous studies^10, 16, 17^ are examples of nucleation via non-classical pathways in geologic materials^2^. Case studies that model nucleation and growth energetics during metamorphism should consider these nucleation pathways and the importance of inherited texture. This may require re-evaluation of the petrographic and 3D textures of minerals in an ostensibly homogeneous matrix, taking account of the possibility that porphyroblast distribution may be controlled to the first order by the availability of specific, energetically favorable, non-randomly distributed nucleation sites including sites of epitaxial nucleation.

Previous models provide important context of how the growth of garnet would be controlled by the distribution and transport of aluminum^36–38^. The seeming importance of epitaxial nucleation in the samples studied here suggests that nucleation is strongly favoured on precursor phases such as muscovite and may be controlled by the distribution of nucleation sites. For garnet growth reactions in which muscovite or any other potential templating phase is also a major reactant, both favourable nucleation sites and a source of Al may be provided, leading to efficient topotaxial overgrowth. It appears here, however, that muscovite provided the nucleation sites but was not a reactant phase, with chlorite likely providing the primary flux of nutrients. Thus the natural rock archive is likely to record a complex interplay between the location of preferable nucleation sites, the location of reactant phases, and the transport properties of nutrient components^37, 39^. For the simple case of samples such as ours, the relative abundance and distributions of precursor chlorite and muscovite may serve as an important control on whether garnet growth is predominantly controlled by nucleation site distribution or the transport of nutrients.

It may also be necessary to consider how epitaxial nucleation may reduce the energetic barrier to garnet crystallization in cases where nucleation is the rate limiting step^24, 40, 41^. This follows from interpretations that garnet crystallization may often be overstepped (i.e. initial growth at *P–T* conditions above than its initial equilibrium stability)^42^. The relative importance of epitaxy and other microtextural relationships as potential controls on macroscale energetics during metamorphism is relatively poorly understood and warrants additional research. Questions remain regarding the absolute energetic contribution that epitaxy might play in modifying the pressure and temperature conditions at which garnet may first grow during prograde metamorphism. It may be necessary to reinterpret metamorphic recrystallization in light of non-classical, specifically epitaxial, nucleation, which may represent a common but generally overlooked process controlling mineral crystallization.

## Methods

### Electron back scattered diffraction (EBSD)

EBSD data were collected on a Tescan MIRA3 LMU Field Emission Gun Scanning Electron Microscope (FEG-SEM) equipped with an Oxford Instruments Symmetry EBSD detector at the Department of Earth and Environmental Sciences at Boston College. Analyses used a 25 kV accelerating voltage and 50–75 nA beam currents, which equates to an angular resolution of 0.7–1.0°^43^. Large area maps of crystallographic texture were produced using Oxford Instruments AZtecHKL acquisition and analysis software (version 4.3). The resulting orientation maps contained 1–4 garnet crystals, which allowed for the complete characterization of garnet crystals and the surrounding minerals. A 1 µm step size was used to achieve a high density of crystallographic solutions within individual grains. This step size is smaller than all individual grains, ensuring > 1 point/grain. Indexing rates for garnet crystals were high (> 95%), commonly resulting in more than 50,000 garnet solutions per map. Indexing rates for muscovite and chlorite were lower, resulting in < 500 solutions per map. Only samples that contained > 100 data points were used for pole figure construction.

EBSD data were analyzed using the MATLAB-based MTEX Toolbox (Version 5.2) (Bachmann et al.^44^). MTEX codes used for this study are available from the author upon request. All data were rotated to a frame of reference with the trace of the rock foliation being horizontal. Individual crystal orientations with median absolute values (M.A.D.) > 0.9 were excluded from the dataset, as they equate to a low confidence in the EBSD solution. Inverse pole figures for garnet were calculated and then contoured for multiples of uniform density (M.U.D.). Pole figures for garnet, chlorite, and muscovite were calculated using the orientation distribution function^45, 46^ such that the mean orientation of each phase was plotted on a lower hemisphere, equal angle pole figure.

### Transmission electron microscopy (TEM)

TEM foil location was determined via optical petrography and Scanning Electron Microscopy (SEM). Foil preparation used a Focused Ion Beam (FIB) liftout on a FEI Helios 600 NanoLab SEM following methodology similar to Wirth (2009), using an oil free high vacuum at Virginia Tech’s Nanoscale Characterization and Fabrication Laboratory (NCFL). The location of the TEM foil was marked by depositing Pt on to the sample to protect the sample from the Ga-ion beam. TEM foils (approximately 2.5 µm × 3.5 µm × 150 nm) were prepared using a Ga-ion beam, with the foil oriented normal to the garnet-chlorite grain boundary. The foil was prepared thicker than common TEM samples to mitigate sample damage in the TEM and ensure a strong diffraction contrast.

TEM analysis used a JEOL2100 TEM operated at 200 kV, with images obtained using a Gatan Ultrascan 1000XP camera. Selected Area Diffraction Patterns (SAED) were taken on ~ 150 nm radius circles. Diffraction patterns were obtained using a Gatan Orius SC200D camera and analyzed using Gatan Digital Micrograph. Electron Dispersive Spectrometry (EDS) scans of the sample utilized the Scanning Transmission Electron Microscopy mode and JEOL EDS Detector.

All crystal structure models were made using VESTA^47^.

## Supplementary Information

Supplementary Information.

Supplementary Movie S1.

Supplementary Movie S2.

Supplementary Movie S3.

## Data Availability

All data is presented in the Supplemental Material. MTeX codes used for this study are available via request to the corresponding author.
