# Supplementary material for: Preferred orientations of garnet porphyroblasts reveal previously cryptic templating during nucleation
Source: Sci Rep. 2021 Mar 25;11:6869. doi: 10.1038/s41598-021-85525-7 (PMC7994400; doi:10.1038/s41598-021-85525-7)
Supplement: Supplementary file 1 — Supplementary Information. [file 41598_2021_85525_MOESM1_ESM.pdf]

Preferred Orientation of Garnet Porphyroblasts Reveals Previously Cryptic Templating During Nucleation

(online Supplementary Material)

Alexandra B. Nagurney*[1], Mark J. Caddick[1], David R.M. Pattison[2], F. Marc Michel[1]

[1] Department of Geosciences, Virginia Tech, Blacksburg VA, USA
[2]Department of Geosciences, University of Calgary, Calgary, Canada
*corresponding author: nagurney@vt.edu

Geographic Coordinates of Samples
08-CW-7A: 11U 0488607, 5467169
08-CW-7.5: 11U 0488662, 5467267
2018PPGrt_01: N 43.1373 W 65.7871

Geologic Maps:
Figure S1: Geologic Map of the Nelson Aureole (redrawn from Pattison and Tinkham, (2009))

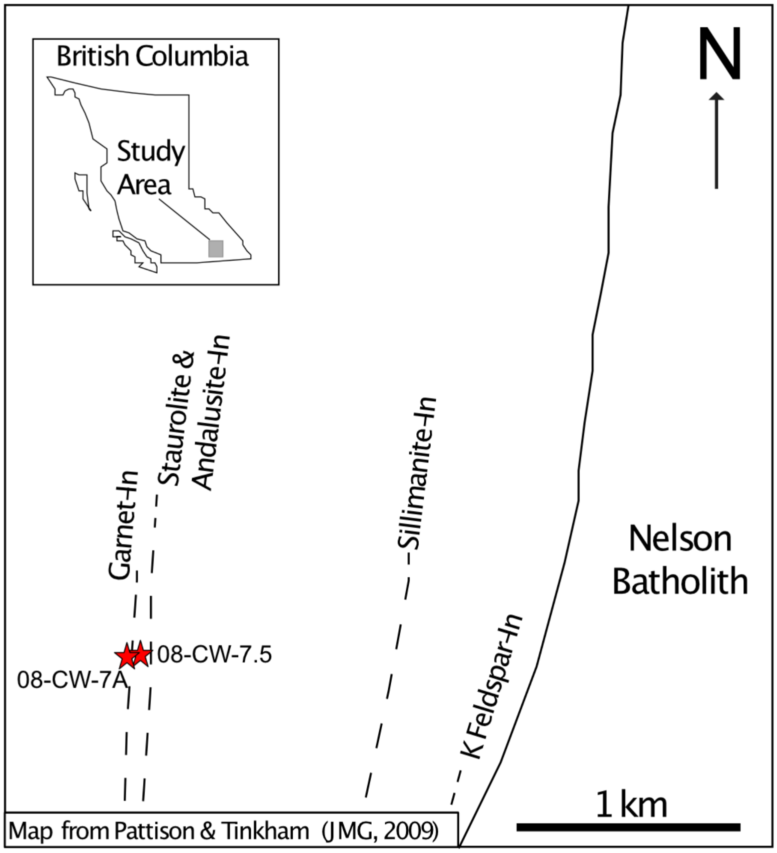

Figure S2: Geologic Map of southwestern Nova Scotia (redrawn from Currie et al., (1998)).

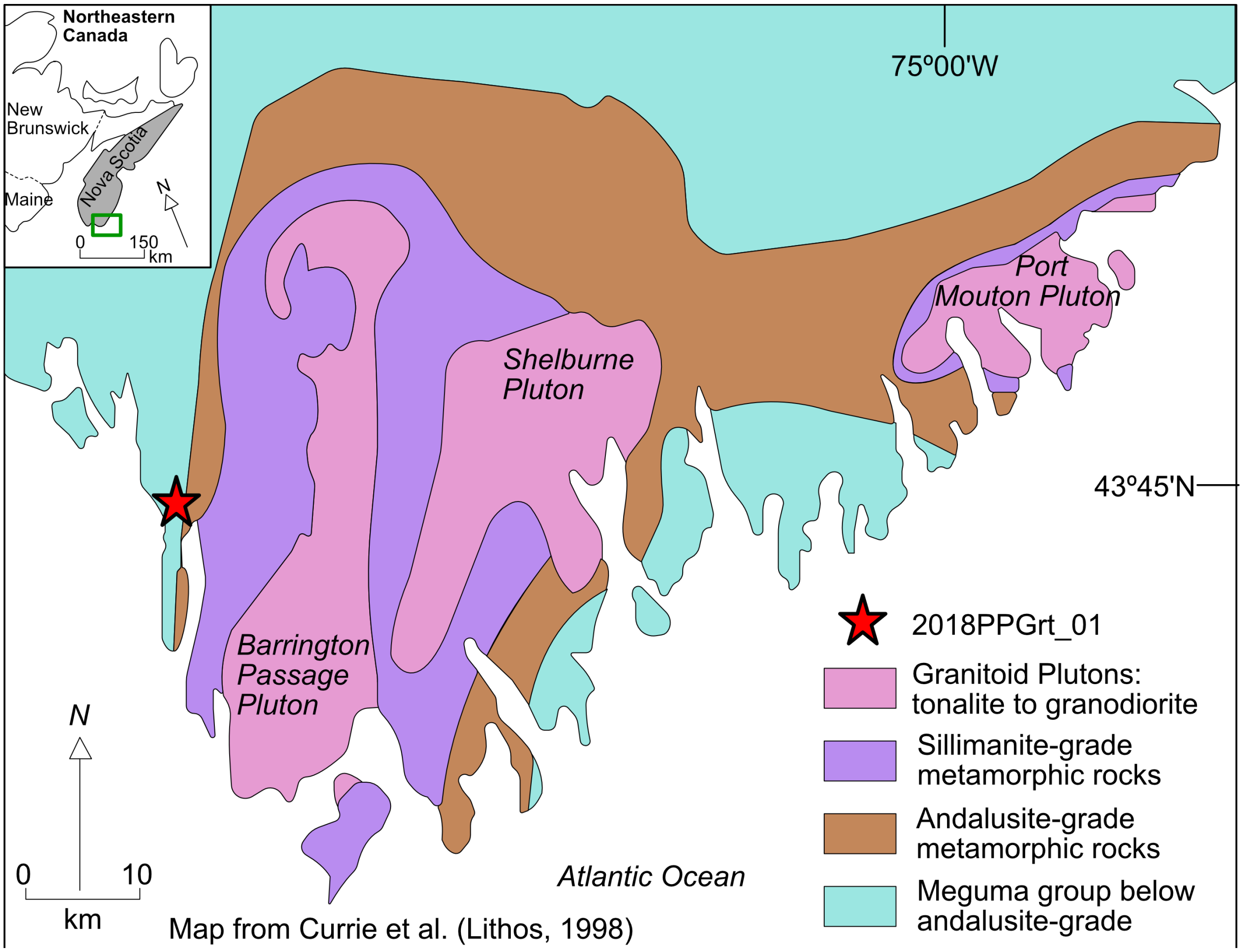

Geologic Background

The Nelson aureole is comprised of graphitic, sulfidic, argillaceous Triassic-Jurassic aged Ymir Group sediments that were regionally metamorphosed to greenschist facies and developed a regional S_1 foliation prior to the intrusion of the Jurassic Nelson batholith. This intrusion further metamorphosed the Ymir Group sediments surrounding the Nelson batholith creating a contact aureole (Pattison and Vogl, 2005). Both 08-CW-7.5 and 08-CW-7A contain biotite, chlorite, garnet, muscovite, plagioclase, and quartz, garnet overgrows the foliation, and peak *P-T* conditions are ~530ºC and 3.5 kbar (Pattison and Vogl, 2005; Pattison and Tinkham, 2009). The Cambrian-aged Mosher's Island formation is a manganese-rich siltstone to slate that was metamorphosed to greenschist-amphibolite facies during the Devonian collision of the Meguma Terrane with North America during the Acadian Orogeny (Schiller and Taylor, 1966; Currie et al., 1998; White and Barr, 2010). Sample 2018PPGrt_01 contains biotite, chlorite, garnet, muscovite, plagioclase, staurolite, and quartz, and garnet overgrows the foliation. In both localities, syn-intrusive garnet growth has previously been interpreted to postdate regional metamorphism (and foliation development) (Taylor and Schiller, 1966; Pattison and Vogl, 2005; Pattison and Tinkham, 2009; White and Barr, 2010).

Figure S3- Petrography of Nelson Aureole and Mosher's Island Formation samples.

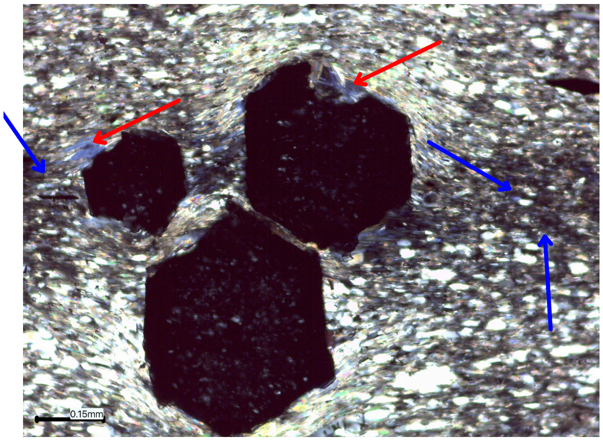

XPL photomicrograph of Mosher's Island formation showing select examples of prograde chlorite (blue arrows) which was identified as being in line with the foliation. Retrograde chlorite (red arrows) is characterized as having a texture in which it appears to be replacing garnet porphyroblasts. Here chlorite is replacing garnet which is breaking down.

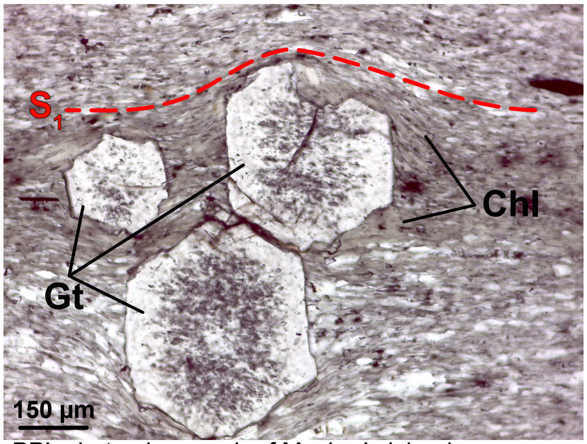

PPL photomicrograph of Mosher's Island formation showing garnet and chlorite. The foliation is horizontal and is deflected by the growth of garnet.

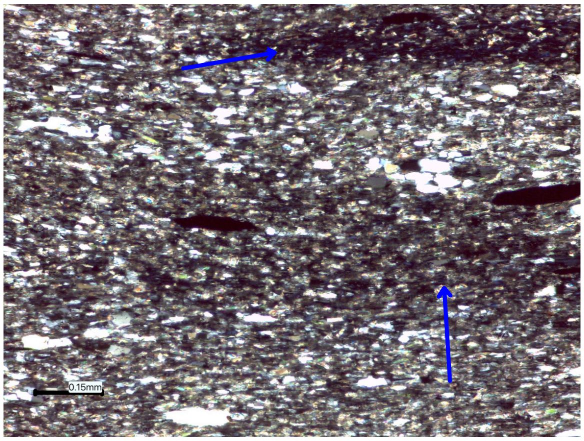

XPL photomicrograph of Mosher's Island formation showing more examples of prograde chlorite (areas highlighted by the blue arrows) in the matrix. Matrix phases include: quartz, plagioclase, chlorite, muscovite, biotite, and ilmenite.

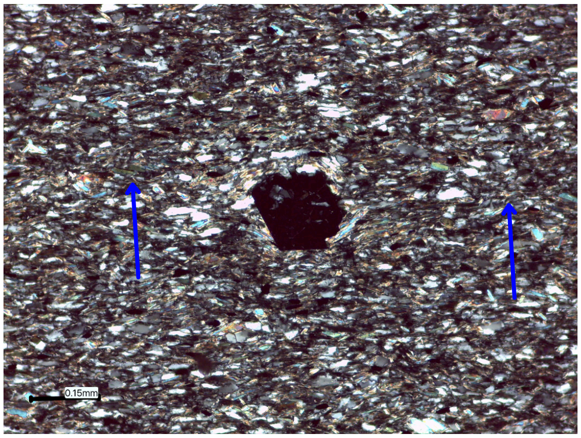

XPL photomicrograph of Nelson aureole sample showing select examples of prograde chlorite (blue arrows) which was identified as being in line with the foliation. There is no clearly identifiable retrograde chlorite in this sample.

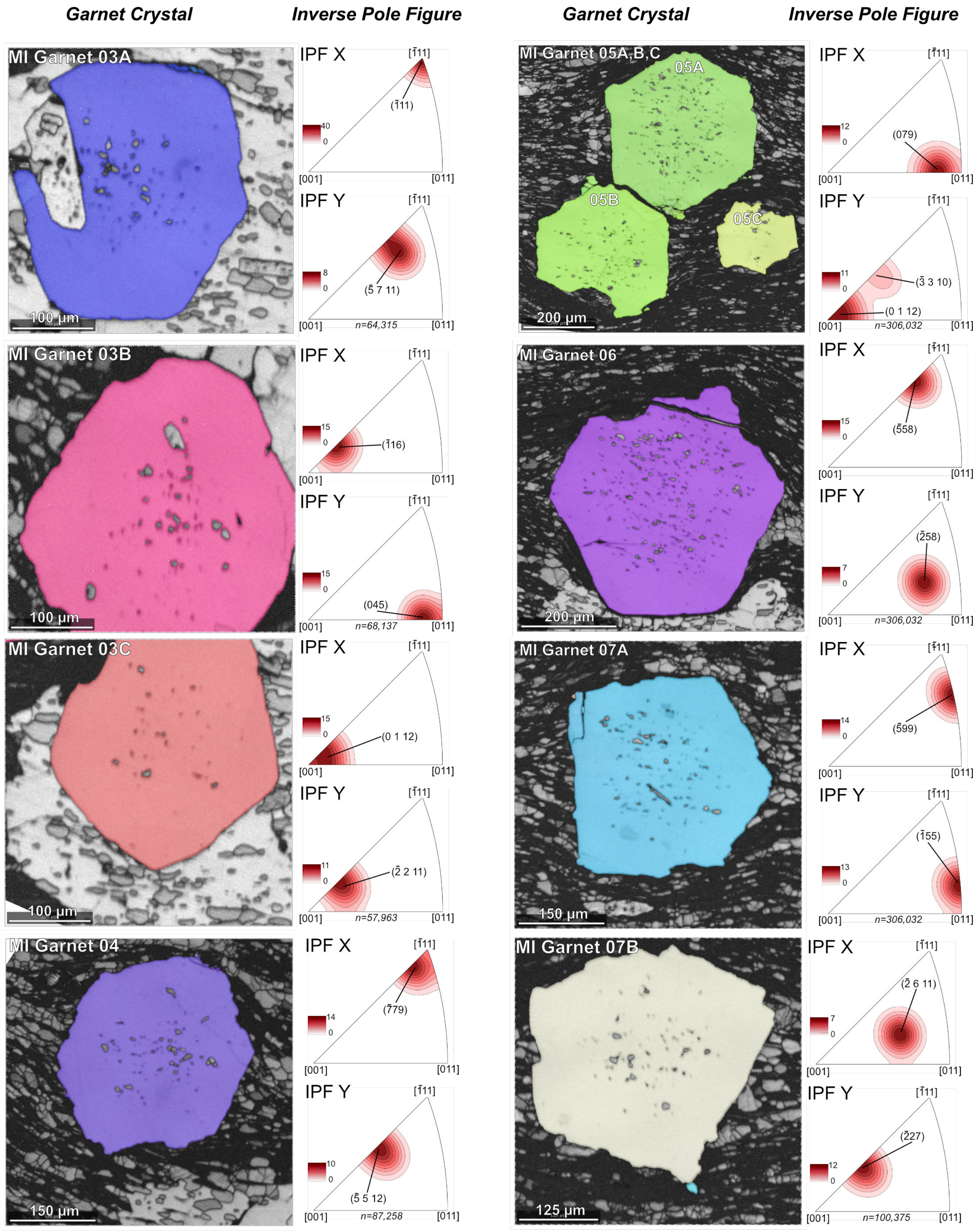

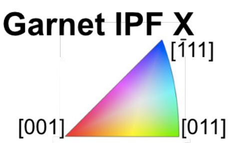

Figure S4: EBSD analyzed garnet crystals color coded for the garnet crystal direction parallel to the X direction (see IPF) and the S1 foliation plotted on the EBSD band contrast image. Inverse pole figures (IPF) X and Y showing the crystal direction of garnet that is parallel to the foliation (X) and perpendicular to the foliation (Y). IPFs are contoured for multiples of uniform distribution. See main text for plotting convention.

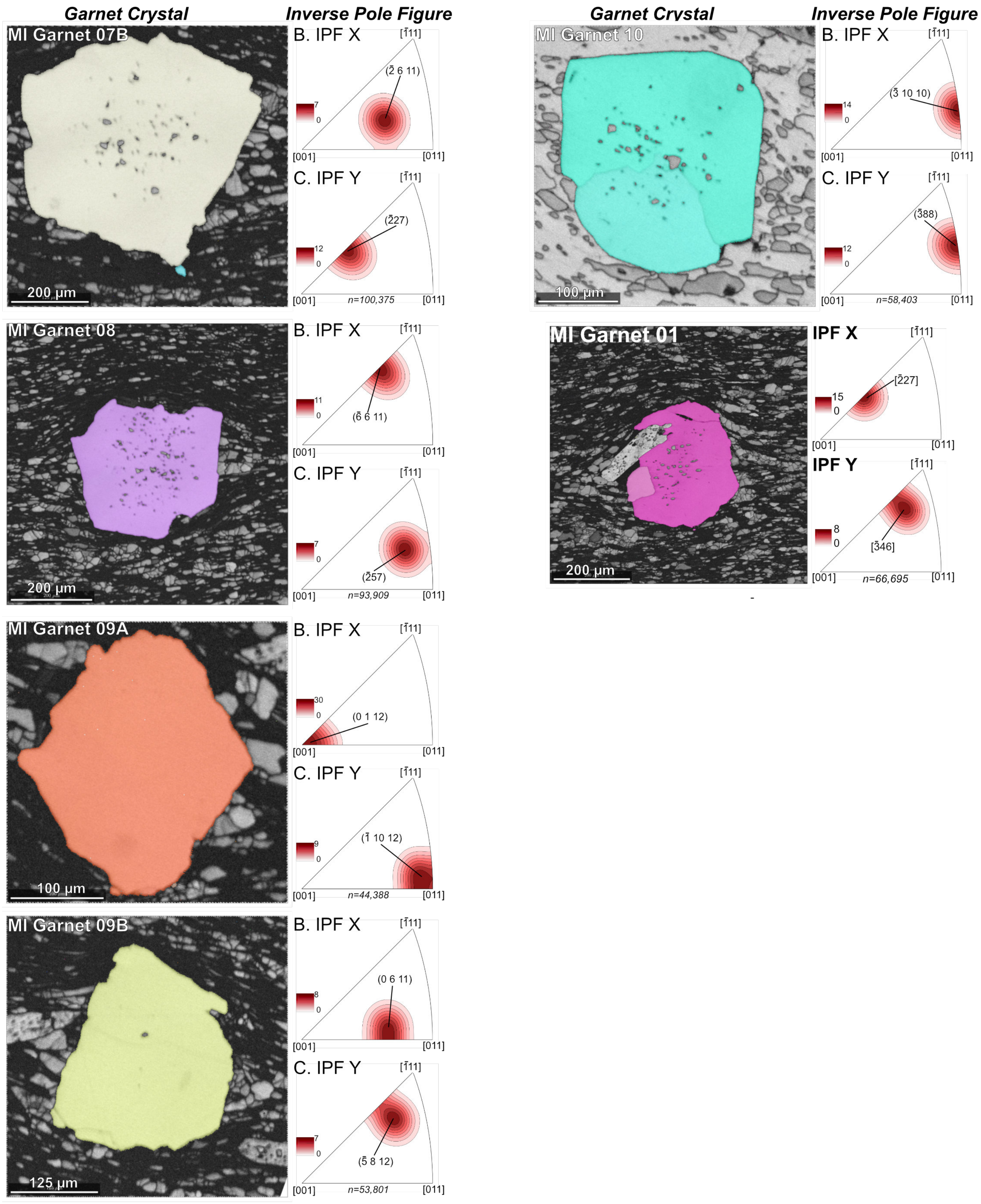

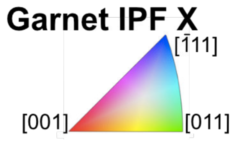

Figure S4 Continued

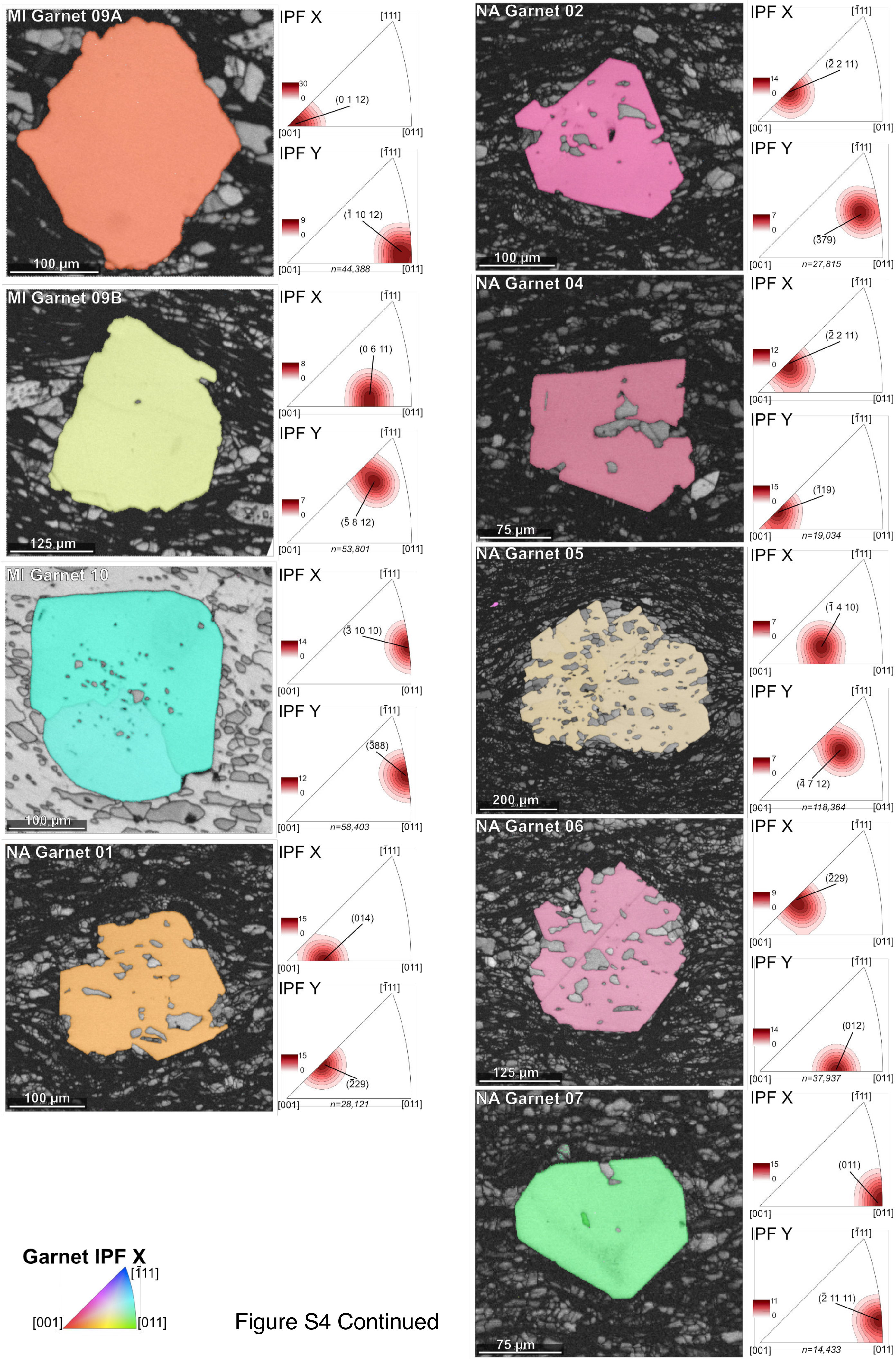

Figure S4 Continued

Table S1: Summary of EBSD results. For each crystal, the corresponding plane to the directions of garnet that are parallel to the X (horizontal, parallel foliation), Y, (vertical, perpendicular to the foliation), and Z (perpendicular to thin section plane, parallel to foliation) are plotted. All crystal planes have been grouped into their corresponding best fit planes (see Fig. 3B). Table shows corresponding crystal plane, crystal plane group, and the angular difference between the two.
The angular difference was calculated using the equation between planes for a cubic crystal like:

$$\cos \Phi = \frac{h_1 h_2 + k_1 k_2 + l_1 l_2}{\sqrt{h_1^2 + k_1^2 + l_1^2} * \sqrt{h_2^2 + k_2^2 + l_2^2}}$$

where Φ = the angle between planes and hkl are the crystal planes in garnet.
The best fit plane was chosen as the mean orientation of all the planes in the cluster.

	X			Y			Z
Garnet Number	Crystal Plane	Crystal Plane Group	Angular Difference (°)	Crystal Plane	Crystal Plane Group	Angular Difference (°)	Crystal Plane
Mosher's Island Formation							
Garnet 01	{-2 2 7}	{-1 1 6}	8.74	{-3 4 6}	{-3 4 6}	0	{0 7 10}
Garnet 03A	{-1 1 1}	{-7 7 9}	7.01	{-5 7 11}	{-3 4 6}	1.97	{-1 10 10}
Garnet 03B	{-1 1 6}	{-1 1 6}	0	{0 4 5}	{-2 11 11}	9.68	{-3 8 10}
Garnet 03C	{0 1 12}	{-1 1 6}	10.45	{-2 2 11}	{-2 2 11}	0	{0 2 9}
Garnet 04	{-7 7 9}	{-7 7 9}	0	{-5 5 12}	{-3 4 6}	10.4	{-1 7 7}
Garnet 05A	{0 7 9}	{0 7 9}	0	{0 1 12}	{-2 2 11}	11.55	{-1 6 8}
Garnet 05B	{0 7 9}	{0 7 9}	0	{-3 4 12}	{-2 2 11}	8.58	{-5 7 9}
Garnet 05C	{0 5 8}	{0 7 9}	5.87	{-1 1 8}	{-2 2 11}	4.4	{-1 3 5}
Garnet 06	{-5 5 8}	{-7 7 9}	6.25	{-2 5 8}	{-3 4 6}	10.74	{-5 11 11}
Garnet 07A	{-5 9 9}	{-7 7 9}	11.94	{-1 5 5}	{-2 11 11}	0.72	{-2 5 12}
Garnet 07B	{-2 6 11}	{0 7 9}	12.94	{-2 2 7}	{-2 2 11}	7.59	{-3 3 7}
Garnet 08	{-6 6 11}	{-7 7 9}	10.08	{-2 5 7}	{-3 4 6}	9.67	{-2 4 9}
Garnet 09A	{0 1 12}	{-1 1 6}	10.45	{-1 10 12}	{-2 11 11}	6.34	{0 5 6}
Garnet 09B	{0 6 11}	{0 7 9}	9.27	{-5 8 12}	{-3 4 6}	3.47	{-6 11 12}
Garnet 10	{-3 10 10}	{0 7 9}	13.89	{-3 8 8}	{-3 4 6}	13.21	{0 1 3}
Nelson Aureole							
7.5 Garnet 01	{0 1 4}	{-1 1 6}	10.39	{-2 2 9}	{-2 2 11}	3.03	{0 1 5}
7.5 Garnet 02	{-2 2 11}	{-1 1 6}	1.15	{-3 7 9}	{-3 4 6}	8.79	{0 4 5}
7.5 Garnet 04	{-2 2 11}	{-1 1 6}	1.15	{-1 1 9}	{-2 2 11}	5.49	{-1 2 9}
7.5 Garnet 05	{-1 4 10}	{-1 1 6}	12.89	{-4 7 12}	{-3 4 6}	7.29	{-3 7 10}
7.5 Garnet 06	{-2 2 9}	{-1 1 6}	4.19	{0 1 2}	{0 1 2}	0	{-3 4 8}

7.5 Garnet 07	{0 1 1}	{0 7 9}	7.13	{-2 11 11}	{-2 11 11}	0	{0 1 12}
7A Garnet 05**	{-1 4 5}	{0 7 9}	8.91	{-7 7 10}	{-7 7 10}	0	{-4 5 10}

** Indicates sample that is shown in the main text.

Figure S5: The following figure shows the EBSD generated phase maps for all 22 analyzed garnet crystals as well as pole figures for garnet, chlorite, and muscovite. All of the garnet crystals indexed very well >1,000 analyses per grain. Most of the chlorite and muscovite crystals indexed well, with a few exceptions. Pole figures are only presented for samples that have >100 points for chlorite and muscovite. For most samples the $(001)_{chl,ms}$ planes are parallel to the trace of the foliation. Where that is not the case, this is due to:

1. All of the orientation data is from one 'oddly oriented' grain that is not representative of the dominant rock foliation (NA Garnet 05).
2. Certain grains were mis indexed: there were a few cases in which staurolite was improperly indexed as muscovite, thus the muscovite pole figure is not representative of the actual muscovite in the rock (MI Garnet 03, MI Garnet 03C).
3. Muscovite or chlorite is an inclusion in ilmenite, thus not representing the dominant orientation of muscovite or chlorite in the rock (MI Garnet 03, MI Garnet 09B).
4. The muscovite and chlorite have been deflected by staurolite (which grew after garnet) and thus does not represent the orientation of chlorite and muscovite during garnet crystallization (MI Garnet 04, MI Garnet 06).

The degree of preferred orientation in the (100) and (010) muscovite and chlorite planes are likely a function of the number of grains analyzed. For example, NA Garnet 04 has a strong preferred orientation for (100) and (010) muscovite, but the associated phase map shows that only 1 muscovite grain was indexed, thus the degree of preferred orientation in those orientations is a function of the number of grains analyzed.

Figure S5

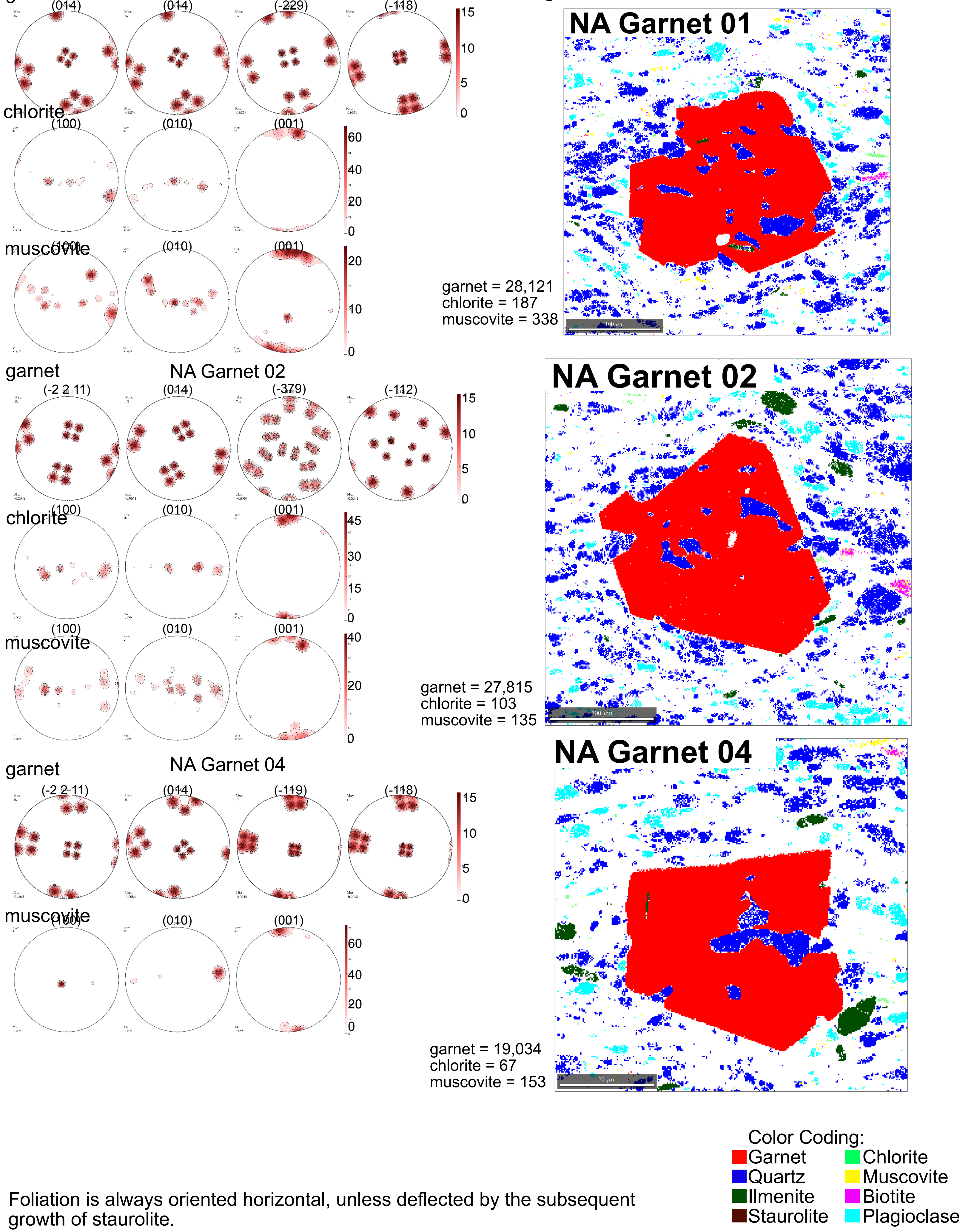

Foliation is always oriented horizontal, unless deflected by the subsequent growth of staurolite.

Figure S5 cont

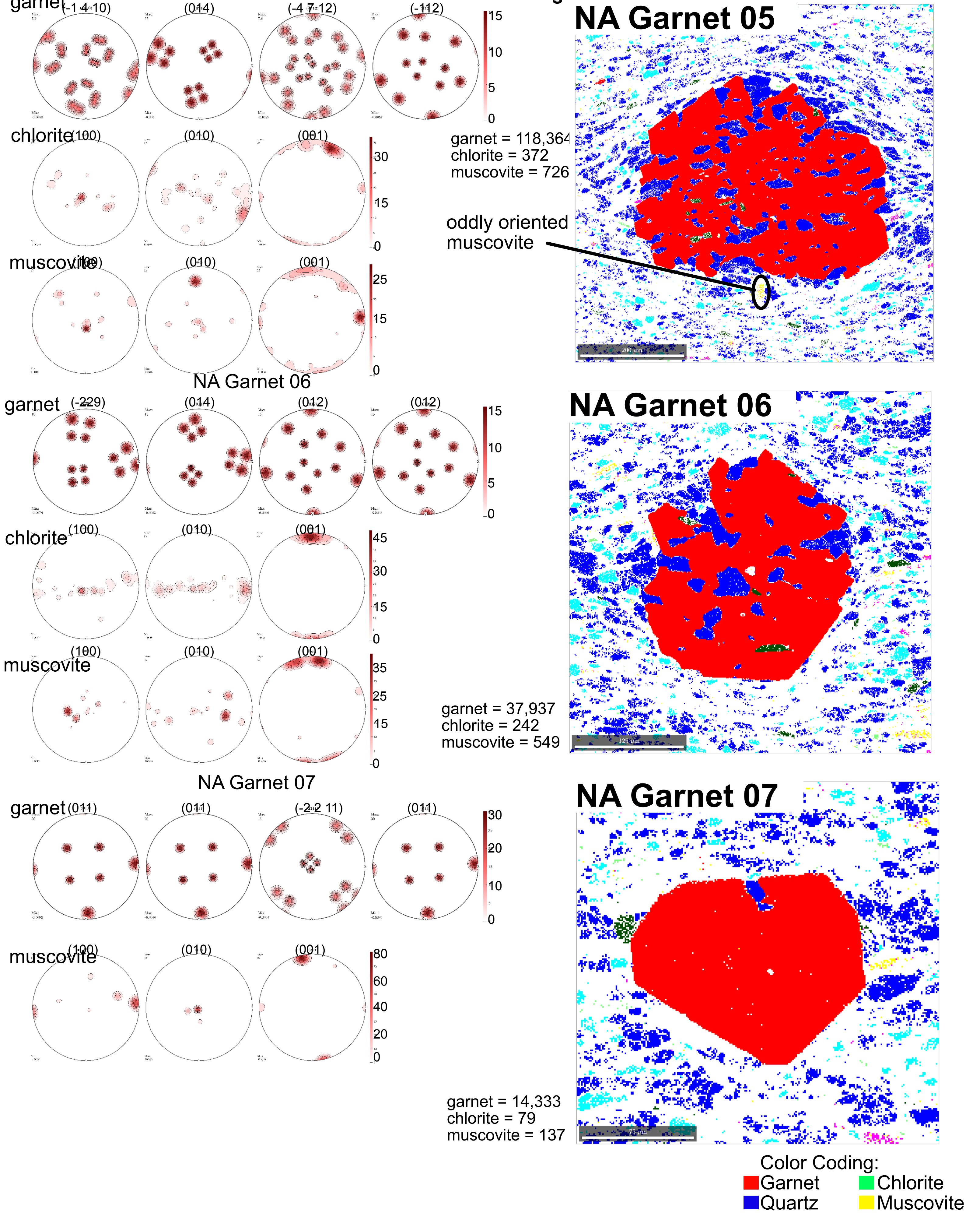

Foliation is always oriented horizontal, unless deflected by the subsequent growth of staurolite.

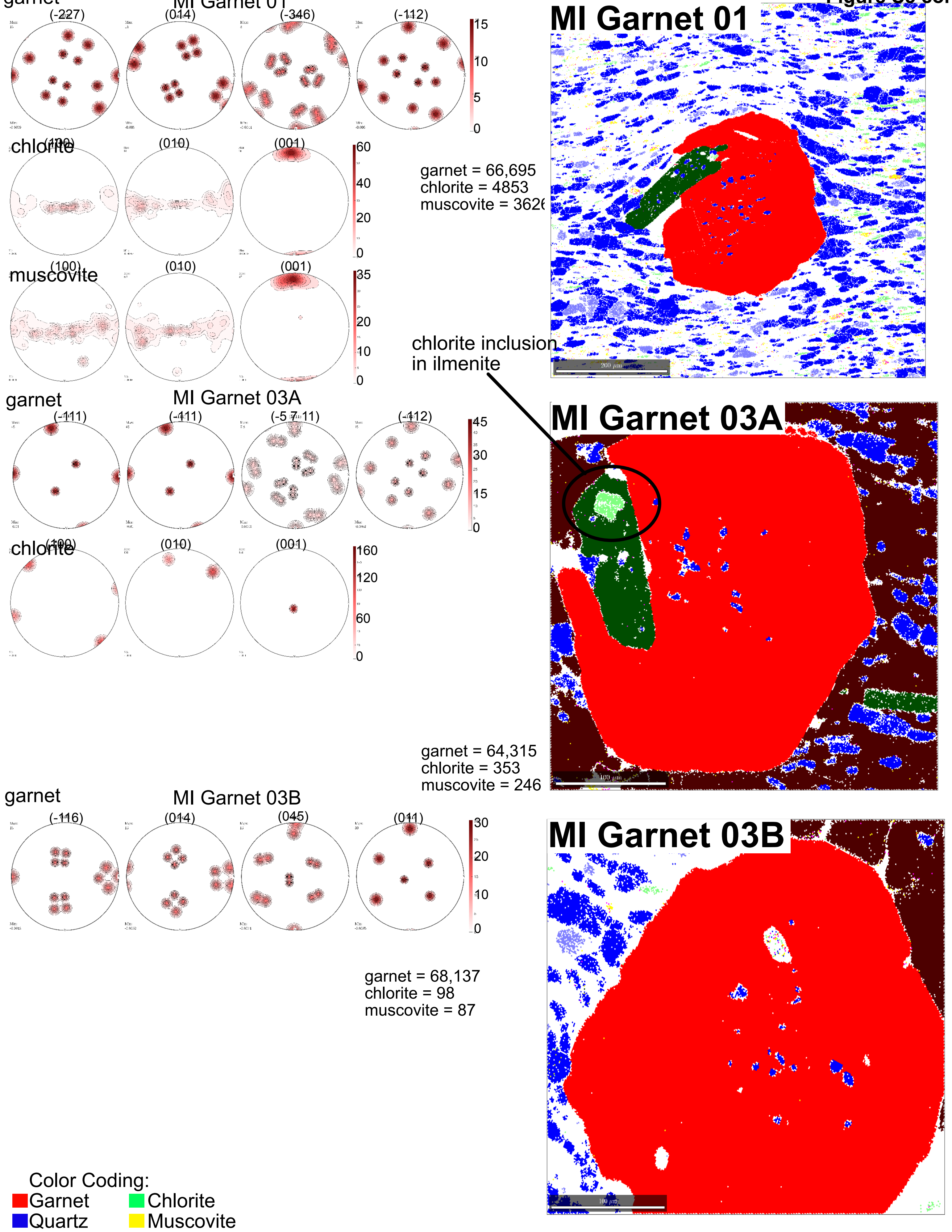

Foliation is always oriented horizontal, unless deflected by the subsequent growth of staurolite.

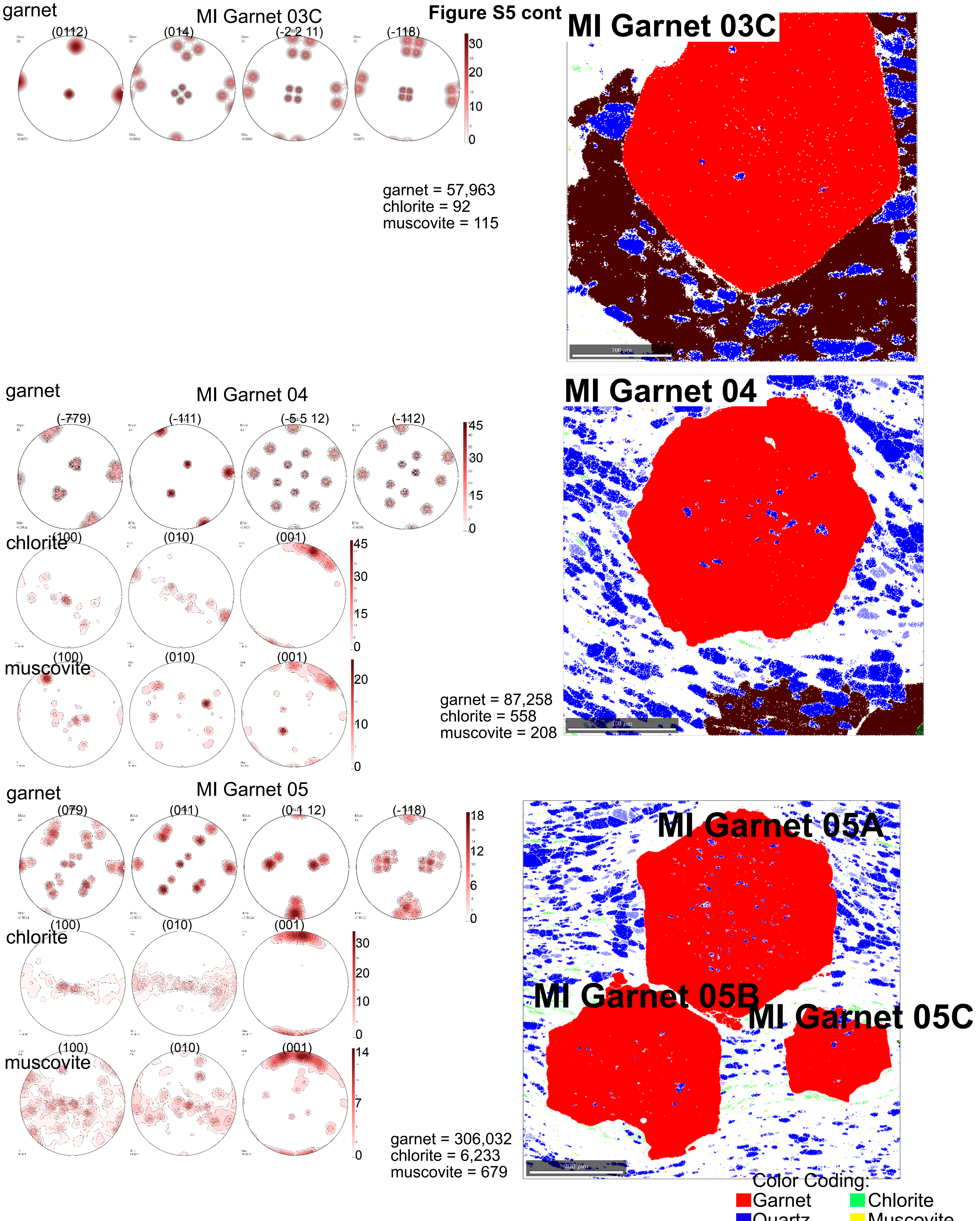

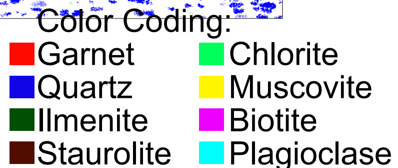

Foliation is always oriented horizontal, unless deflected by the subsequent growth of staurolite.

Figure S5 cont

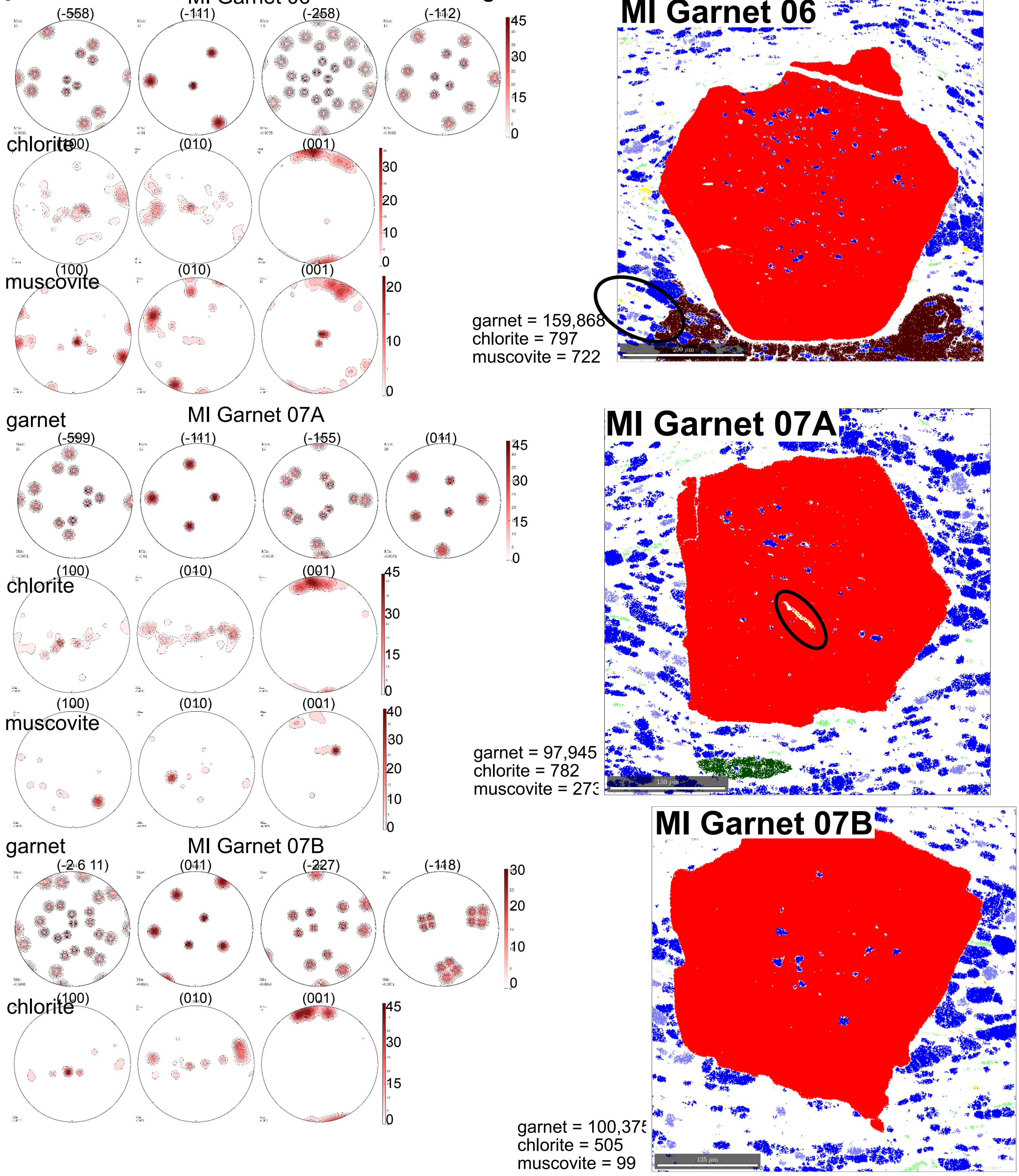

Color Coding:
Garnet
Chlorite
Quartz
Muscovite
Ilmenite
Biotite
Staurolite
Plagioclase

Foliation is always oriented horizontal, unless deflected by the subsequent growth of staurolite.

Figure S5 cont

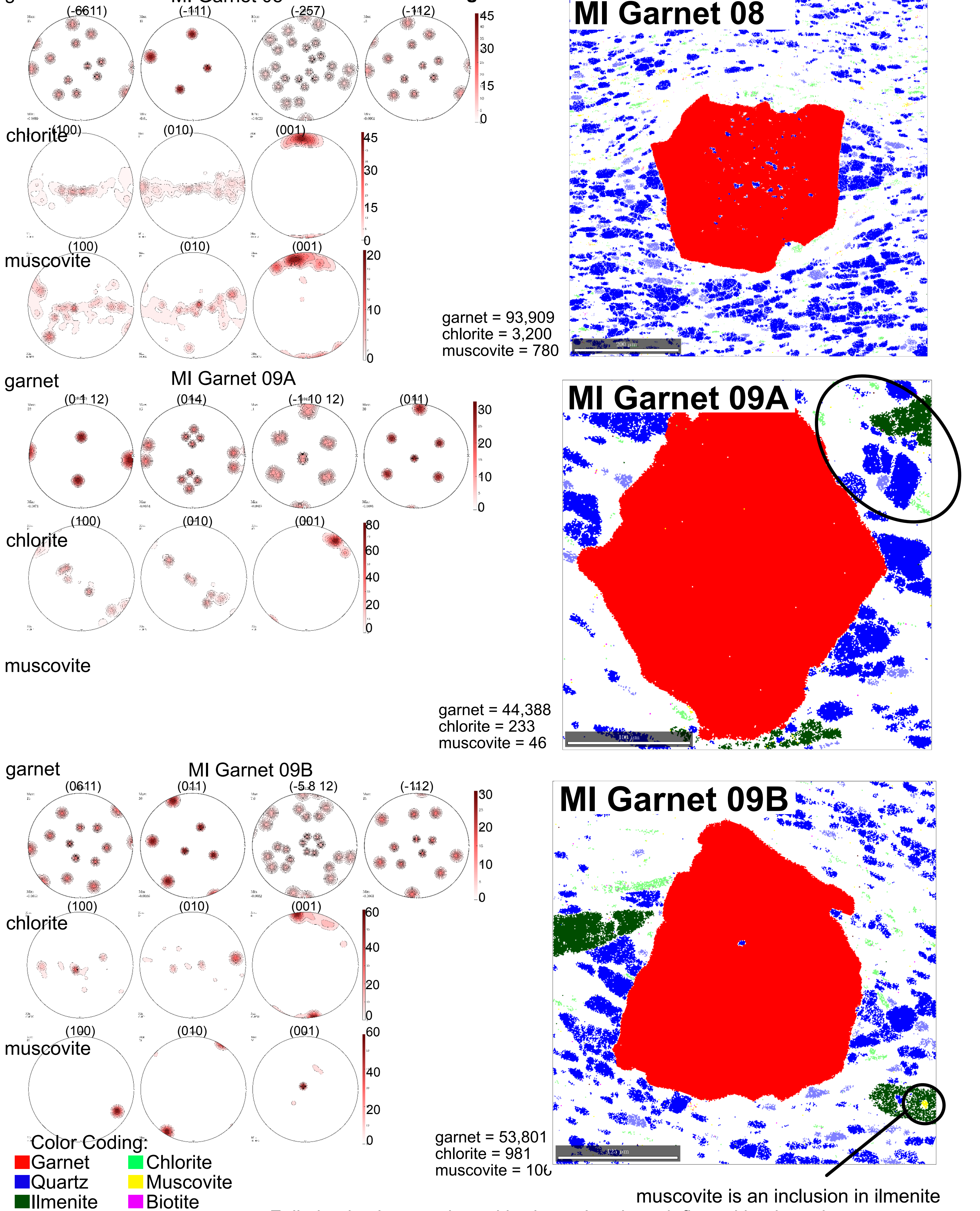

Foliation is always oriented horizontal, unless deflected by the subsequent growth of staurolite.

Figure S5 cont

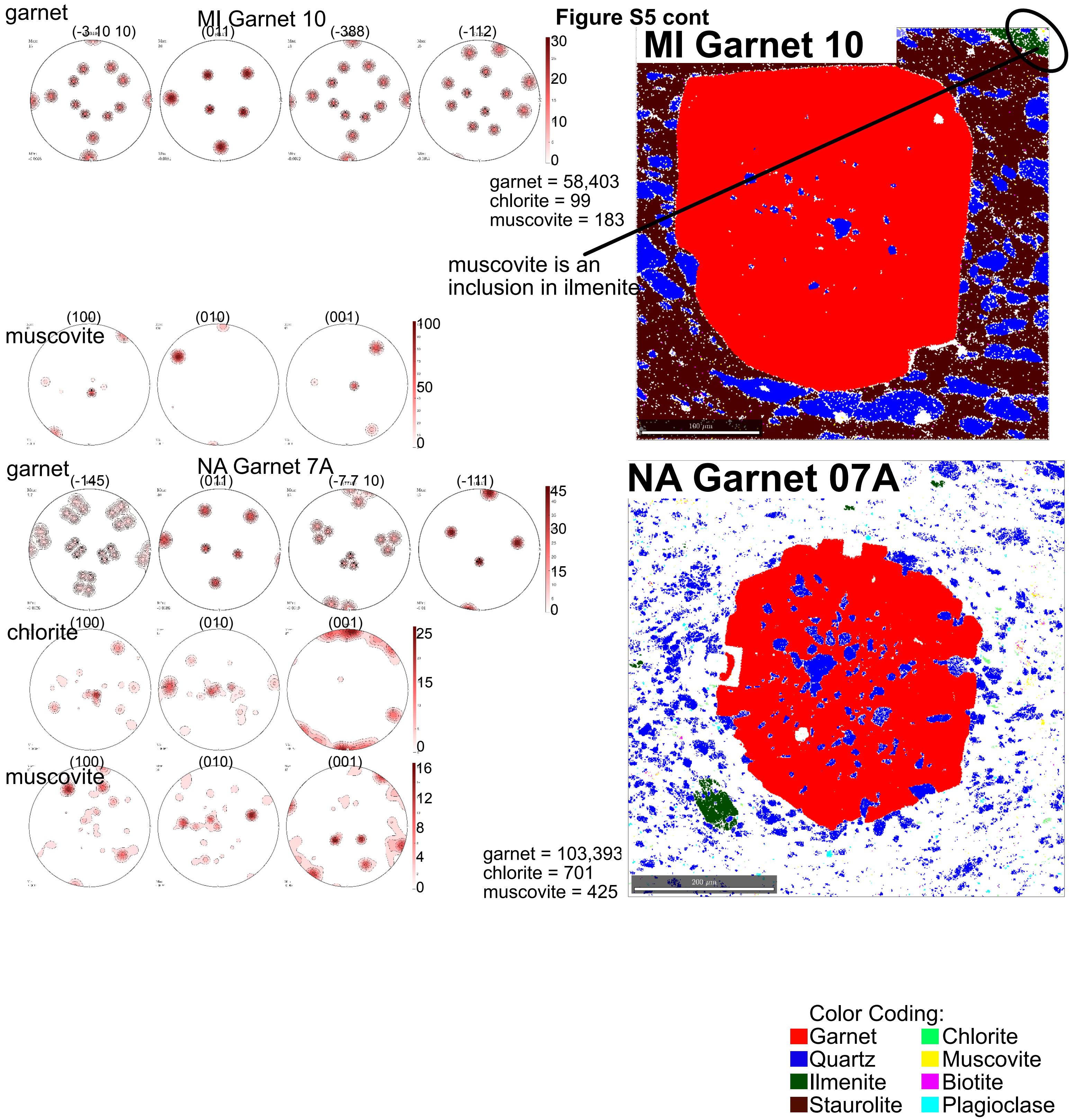

Foliation is always oriented horizontal, unless deflected by the subsequent growth of staurolite.

Figure S6- SEM images of FIB liftout

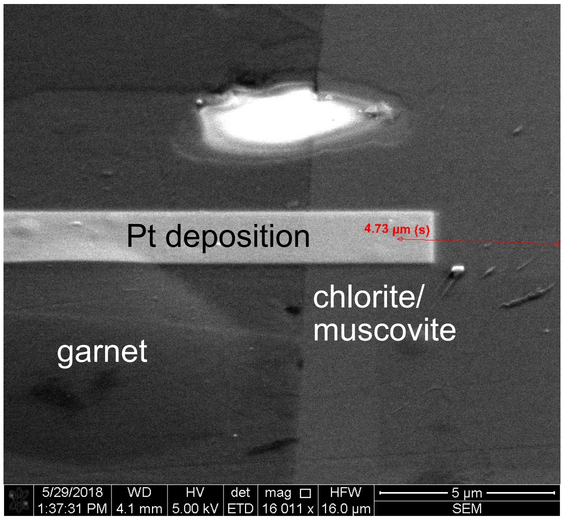

SEM image of the TEM foil location. Sample orientation is the same as above.

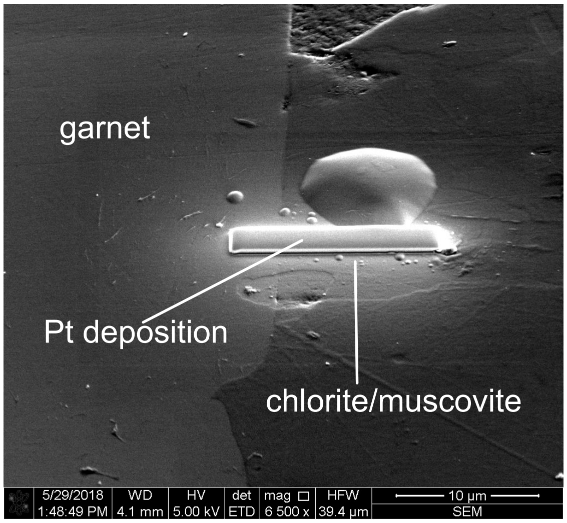

SEM image of the TEM foil location. Sample is tilted at an angle to show the Pt deposition (which is the exact location of the TEM foil).

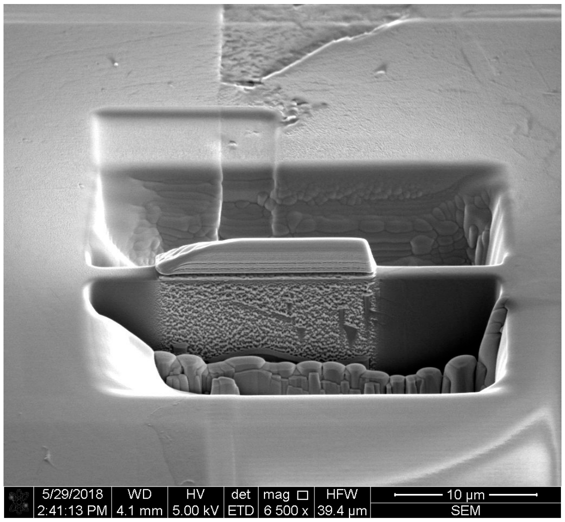

SEM image of the TEM foil location. Sample is tilted at an angle to show the Pt deposition (which is the exact location of the TEM foil).

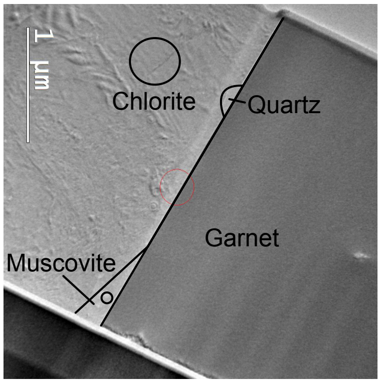

Overview of TEM sample showing locations noted below.

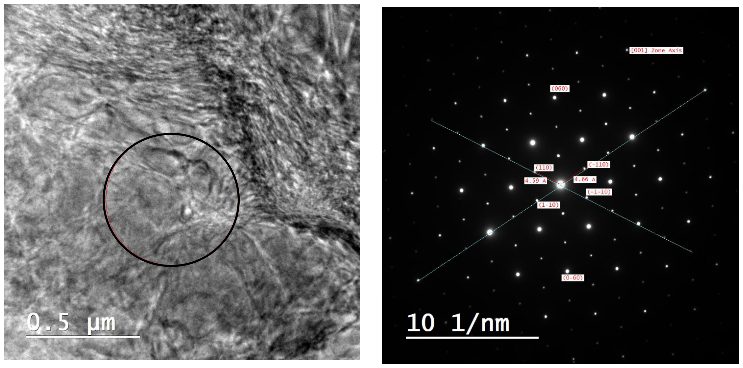

Chlorite diffraction pattern (right)
and location of diffraction pattern (left).

Indexed planes show this is oriented
parallel to the [001] zone axis.

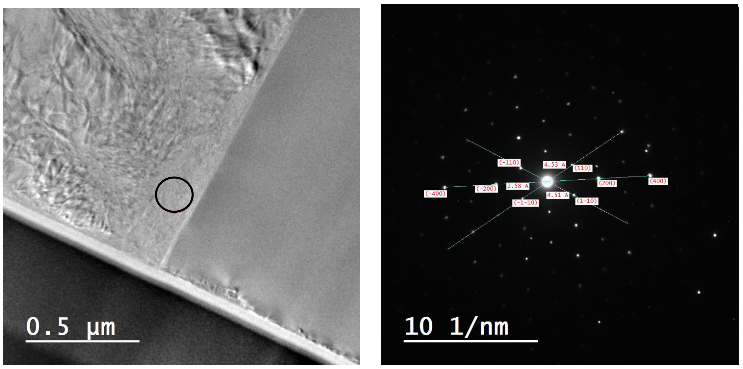

Muscovite diffraction pattern (right)
and location of diffraction pattern (left).

Indexed planes show this is oriented
parallel to the [001] zone axis.

Figure S7- Top: overview of TEM sample showing locations of chlorite and muscovite diffraction patterns. The sample was not tilted between collecting the chlorite and muscovite diffraction patterns. They are both in the same orientation, looking down the [001] zone axis.

Video S1: 3D orientation of muscovite and garnet for the $(079)_{gt}$ orientation.
Video S2: 3D orientation of muscovite and garnet for the $(\bar{7}79)$ orientation.
Video S3: 3D orientation of muscovite and garnet for the $(\bar{1}16)$ orientation.

References

Currie, K.L., Whalen, J.B., Davis, W.J., Longstaffe, F.J., Cousens, B.L., 1998. Geochemical evolution of peraluminous plutons in southern Nova Scotia, Canada-a pegmatite-poor suite. Lithos 44, 117–140. https://doi.org/10.1016/S0024-4937(98)00051-6

Pattison, D.R.M., Tinkham, D.K., 2009. Interplay between equilibrium and kinetics in prograde metamorphism of pelites: an example from the Nelson aureole, British Columbia. Journal of Metamorphic Geology 27, 249–279. https://doi.org/10.1111/j.1525-1314.2009.00816.x

Pattison, D.R.M., Vogl, J.J., 2005. Contrasting sequences of metapelitic mineral-assemblages in the aureole of the tilted Nelson Batholith, British Columbia: Implications for phase equilibria and pressure determination in andalusite-sillimanite-type settings. Canadian Mineralogist 43, 51–88. https://doi.org/10.2113/gscanmin.43.1.51

Taylor, F., Schiller, E., 1966. Metamorphism of the Meguma group of Nova Scotia. Canadian Journal of Earth Sciences 3, 959–974.

White, C.E., Barr, S.M., 2010. Lithochemistry of the Lower Paleozoic Goldenville and Halifax groups, southwestern Nova Scotia, Canada: Implications for stratigraphy, provenance, and tectonic setting of the Meguma terrane. Geological Society of America Memoir 206, 347–366. https://doi.org/10.1130/2010.1206(15).
